# Pharmacological Interventions for Orthostatic Hypotension: A Systematic Review

**DOI:** 10.7759/cureus.89911

**Published:** 2025-08-12

**Authors:** Anushka Verma, Eiman Saraya, Mehjabin S Haque, Mithum Senaratne, Safiyyah Khan, Alousious Kasagga, Iana A Malasevskaia

**Affiliations:** 1 Internal Medicine, Smt. Nathiba Hargovandas Lakhmichand (NHL) Municipal Medical College, Ahmedabad, IND; 2 Internal Medicine, Saint Martinus University, Willemstad, CUW; 3 Medicine, Jiangxi University of Traditional Chinese Medicine, Nanchang, CHN; 4 Internal Medicine, University of Ruhuna, Matara, LKA; 5 Internal Medicine, Basaveshwara Medical College & Hospital, Chitradurga, IND; 6 Pathology, Peking University, Beijing, CHN; 7 Principles and Practice of Clinical Research (PPCR), Harvard T.H. Chan School of Public Health, Boston, USA

**Keywords:** atomoxetine, droxidopa, fludrocortisone, midodrine, octreotide, orthostatic hypotension, pharmacological interventions, pyridostigmine, systematic review

## Abstract

Orthostatic hypotension (OH), defined as a sustained drop in systolic (≥20 mmHg) or diastolic (≥10 mmHg) blood pressure upon standing, is a debilitating condition prevalent in older adults and individuals with neurodegenerative disorders. It significantly impacts quality of life, leading to dizziness, falls, and syncope, and is associated with increased morbidity and mortality. This systematic review evaluates the efficacy and safety of pharmacological treatments for OH. Following the PRISMA 2020 guidelines, 25 studies, including randomized (RCTs) and non-randomized controlled trials (NRCTs), were analyzed. Study quality was assessed using the Cochrane Risk of Bias 2 (ROB 2) tool, the Joanna Briggs Institute (JBI) Checklist, and the Newcastle-Ottawa Scale (NOS). The Grading of Recommendations, Assessment, Development and Evaluation (GRADE) framework was applied to evaluate the certainty of evidence across key outcomes. Drugs approved by the U.S. Food and Drug Administration (FDA), such as droxidopa and midodrine, consistently improve orthostatic symptoms and are recommended as first-line therapies. Atomoxetine and fludrocortisone showed moderate efficacy, while pyridostigmine in combination therapies provided additional benefits. Octreotide demonstrated potential for refractory OH but lacked robust evidence. Adverse effects, including supine hypertension, dizziness, gastrointestinal disturbances, and fatigue, highlight the need for personalized therapy to balance efficacy and tolerability. While pharmacological treatments show promise, further comparative and long-term studies are necessary to refine therapeutic strategies and improve patient outcomes.

## Introduction and background

Orthostatic hypotension (OH), also known as postural hypotension, is a prevalent condition characterized by a significant drop in blood pressure upon standing. It is defined as a decrease in systolic blood pressure (SBP) of at least 20 mmHg or diastolic blood pressure (DBP) of at least 10 mmHg within three minutes of standing or tilting the head to a 60-degree angle on a tilt table [1]. The condition becomes increasingly common with age due to physiological declines in baroreceptor sensitivity and the rising incidence of autonomic neurodegenerative diseases among older adults [2]. OH affects approximately 5% of individuals under 50 years of age, while its prevalence escalates to around 30% in those over 70 years [3,4]. Hospitalized patients are particularly vulnerable, with prevalence rates reaching as high as 60% in certain clinical settings [4,5]. Additionally, more than 30% of individuals with diabetes mellitus and similar proportions among patients with Parkinson's disease (PD) experience OH [6,7].

OH can be classified into two categories based on its pathophysiology: neurogenic OH (NOH) and non-neurogenic OH. NOH results from impaired baroreflex-mediated vasoconstriction due to dysfunction in the autonomic nervous system, caused by neurodegenerative diseases (e.g., PD, multiple system atrophy), diabetic autonomic neuropathy, spinal cord injuries, and autoimmune disorders such as pure autonomic failure [8]. In contrast, non-neurogenic OH arises from alterations in blood volume or pressure regulation, often due to dehydration, blood loss, certain medications, or conditions that compromise cardiac pump function [2,8].

Elderly patients with OH are particularly susceptible to symptoms such as falls, dizziness, and syncope [9]. These symptoms can lead to functional impairment, head injuries, bone fractures, and increased hospitalization rates; up to 55% of institutionalized individuals experience these complications [10]. Patients may also report fatigue, weakness, blurry vision, cognitive decline, and a specific type of headache known as "coat hanger headache," typically alleviated by lying down [2]. Notably, 59% of patients with NOH report a negative impact on their quality of life due to associated symptoms, underscoring the significant burden this condition places on the elderly population [11].

Effective management of OH is crucial, as untreated or poorly controlled cases can result in debilitating symptoms, diminished quality of life, and an increased relative risk of all-cause mortality by up to 50% [12]. While non-pharmacological interventions, such as lifestyle modifications and physical maneuvers, are important first-line strategies, pharmacological treatment is often necessary for severe or resistant cases [13]. Several medications, including FDA-approved droxidopa and midodrine, along with other agents such as pyridostigmine, atomoxetine, and fludrocortisone, have demonstrated efficacy in improving OH symptoms in various clinical trials [14-38]. However, the optimal pharmacological approach remains a subject of debate, as treatment responses can vary significantly based on factors such as the underlying cause of OH, comorbidities, and concurrent medications.

This systematic review aims to evaluate and synthesize the current evidence on pharmacological interventions for managing OH. By examining randomized controlled trials (RCTs), cohort studies, and other relevant clinical research, this review seeks to assess the reported efficacy and safety of various pharmacological treatments, explore the comparative benefits of different agents, and provide insights into optimizing treatment strategies for individuals suffering from this condition. Additionally, we applied the Grading of Recommendations, Assessment, Development and Evaluation (GRADE) [39] framework to assess the overall certainty of evidence across outcomes.

## Review

Methods

Study Design

This systematic review was conducted following the PRISMA 2020 guidelines [40], focusing on pharmacological interventions for OH. The review included studies that employed various designs, including RCTs, controlled clinical trials (CCTs), clinical studies, and observational studies. The aim was to evaluate both the efficacy and safety of these interventions in adult populations diagnosed with OH.

Eligibility Criteria

This systematic review included original studies published in English that involved adults aged 18 years or older diagnosed with OH caused by neurogenic conditions. These conditions included pure autonomic failure, multiple system atrophy, PD, diabetes mellitus, and idiopathic OH. The review focused on studies that compared the efficacy of pharmacological interventions, specifically droxidopa, midodrine, atomoxetine, pyridostigmine, fludrocortisone, and octreotide, against placebo or other pharmacological treatments. Only high- and moderate-quality studies were considered, including RCTs, CCTs, and observational studies such as cohort studies, case-control studies, and cross-sectional studies.

Exclusion criteria for this review included studies involving individuals under the age of 18, as well as those focused solely on non-pharmacological treatments for OH. Additionally, studies that did not report results or were incomplete, such as ongoing trials without published outcomes, were excluded. Research involving healthy individuals or participants with OH resulting from acute medical conditions, such as acute hemorrhage, severe dehydration, or conditions requiring hemodialysis, was also omitted. This exclusion ensured that the review focused on chronic neurogenic causes of OH rather than transient or acute episodes. Furthermore, non-original studies, such as reviews, meta-analyses, editorials, commentaries, case reports, case series, and posters as well as animal studies, were not included in this review.

Data Collection

Data collection was performed through a systematic literature search across multiple databases, including PubMed/Medline, Cochrane Library, ScienceDirect, Europe PubMed Central, ClinicalTrials.gov, and the International Clinical Trials Registry Platform (ICTRP). The search was conducted from November 15 to November 16, 2024, utilizing a combination of keywords and MeSH terms related to OH and pharmacological treatments.

Search Strategy

The search strategy involved a comprehensive approach to ensure the retrieval of relevant studies. Keywords included "Orthostatic hypotension", "Midodrine", "Droxidopa", "Fludrocortisone", "Octreotide", "Atomoxetine", and "Pyridostigmine". The search utilized both MeSH terms and free-text keywords to maximize the identification of pertinent literature (Appendices).

Screening and Selection Process

After the initial search, the retrieved articles were imported into the Rayyan app for screening [41]. Two independent reviewers assessed the titles and abstracts for eligibility based on the predefined criteria. Full texts of potentially relevant studies were then reviewed to confirm eligibility. Disagreements between reviewers were resolved through discussion to ensure a rigorous selection process.

Data Extraction and Management

Data extraction was conducted using a standardized form developed to capture essential information from each study. This included author details, year of publication, study design, participant demographics, intervention specifics, outcomes measured, and results. The data extraction process was performed independently by two reviewers, with discrepancies addressed through consensus.

Quality Appraisal Based on the Design of the Included Studies

Quality appraisal of the included studies was conducted using appropriate tools based on study design. For RCTs, the Cochrane Risk of Bias (ROB 2) tool [42] was utilized to assess the risk of bias, Joanna Briggs Institute (JBI) checklist [43] for quasi-experimental studies (non-randomized experimental studies), while the Newcastle-Ottawa Scale (NOS) [44] was applied to evaluate the quality of observational studies. This appraisal aimed to determine the methodological rigor and reliability of the findings presented in the included studies.

Data Synthesis

Data synthesis involved a narrative analysis of the findings from the included studies, focusing on the efficacy and safety of the pharmacological interventions. Results were organized into tables to facilitate comparison across studies, highlighting key outcomes such as changes in orthostatic blood pressure and reported adverse effects. Due to heterogeneity in study populations, intervention protocols, and primary outcome measures, meta-analysis was not feasible. Instead, we present a structured narrative synthesis grouped by pharmacologic agent and by outcome. The certainty of evidence for each outcome was subsequently assessed using the GRADE [39] framework, which rates the quality of evidence as “high,” “moderate,” “low,” or “very low.” GRADE assessments were based on five domains: risk of bias, inconsistency, indirectness, imprecision, and publication bias.

Results

Study Selection

**Figure 1:**
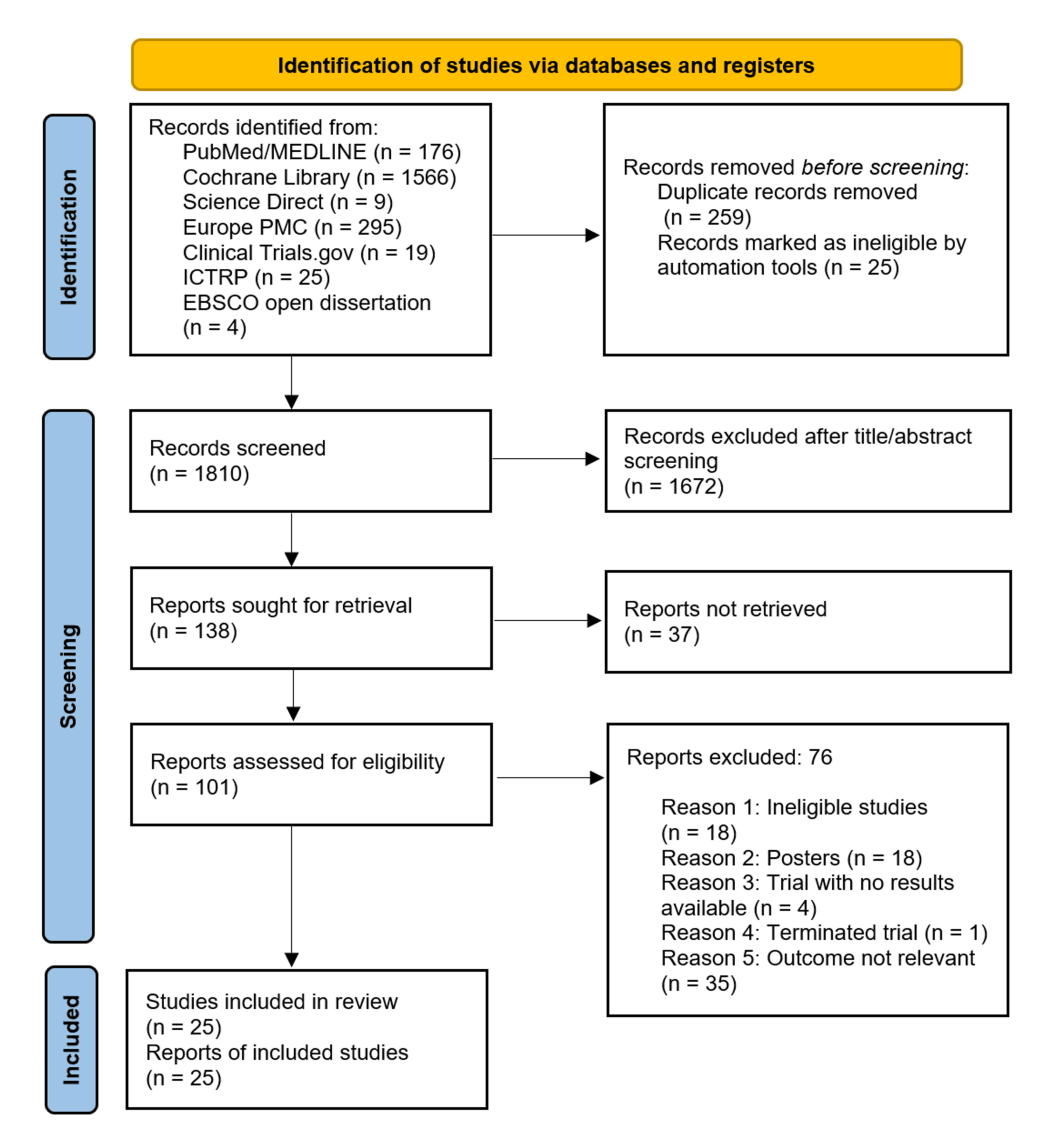
PRISMA flow diagram PRISMA: Preferred Reporting Items for Systematic Review and Meta-Analysis, MEDLINE: Medical Literature Analysis and Retrieval System Online, PMC: PubMed Central, ICTRP: International Clinical Trials Register Platform, EBSCO: Elton B. Stephens Company.

A thorough search strategy retrieved 2,094 initial records from various databases and registers, using relevant filters. After removing duplicates and screening by title and abstract, full-text articles were chosen for further assessment. Ultimately, 25 studies were included in the final review. The study selection process is visually represented in a PRISMA flow diagram (Figure 1).

Risk of Bias Assessment

**Figure 2:**
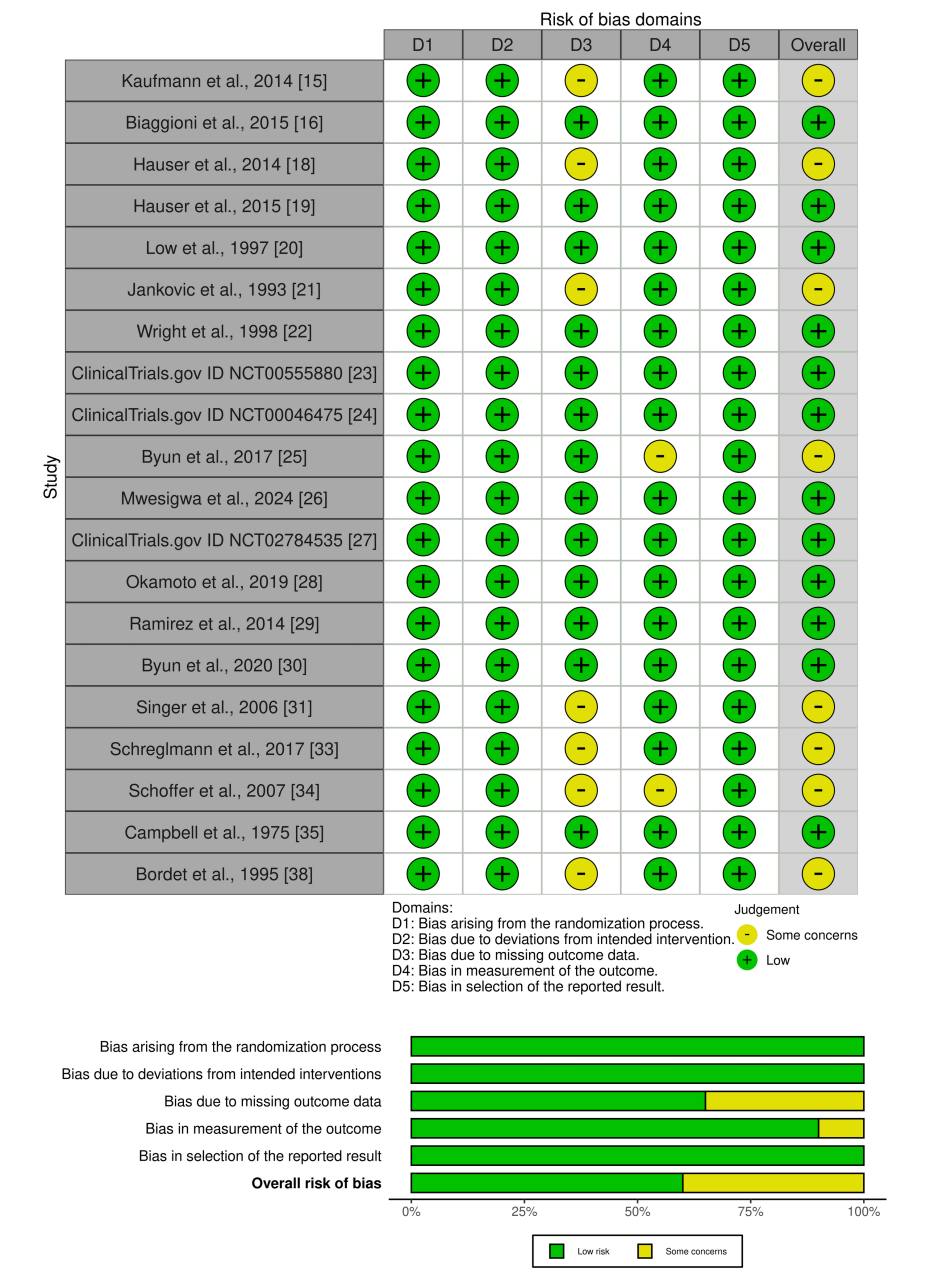
Summary of risk of bias using Cochrane RoB 2 tool and risk of bias graph generated with the Robvis tool RoB 2: risk of bias tool 2, Robvis: risk of bias visualization tool. Source: [15,16,18-35,38].

The risk of bias in the 25 studies included was assessed using appropriate tools for each study design. The Cochrane RoB 2 tool [42], was used to evaluate the quality of the 20 RCTs, with the results visually displayed using the risk of bias visualization (Robvis) tool [45], as shown in Figure 2. Most RCTs were categorized as having a low risk of bias, although a few raised concerns regarding incomplete outcome data or potential bias in outcome measurement. However, the overall risk of bias was considered acceptable for inclusion in the final review.

As outlined in Table 1, the JBI critical appraisal tool [43] was applied to non-randomized interventional studies, with most studies determined to be of good quality. Given the absence of high-risk or low-quality studies, all studies were included in the final analysis to strengthen the overall confidence in the findings.

**Table 1:** JBI critical appraisal checklist for quasi-experimental studies (non-randomized experimental studies) JBI: Joanna Briggs Institute. D: domain. D1: Is it clear in the study what is the "cause" and what is the "effect" (i.e. there is no confusion about which variable comes first)? D2: Were the participants included in any comparisons similar? D3: Were the participants included in any comparisons receiving similar treatment/care, other than the exposure or intervention of interest? D4: Was there a control group? D5: Were there multiple measurements of the outcome both before and after the intervention/exposure? D6: Was follow-up complete and if not, were differences between groups in terms of their follow-up adequately described and analyzed? D7: Were the outcomes of participants included in any comparisons measured in the same way? D8: Were outcomes measured in a reliable way? D9: Was appropriate statistical analysis used?

Study	D1	D2	D3	D4	D5	D6	D7	D8	D9	JBI score/overall quality
Hauser et al. (2022) [14]	Yes	Yes	Yes	Yes	Yes	Yes	Yes	Yes	Yes	9 out of 9/good quality
Isaacson et al. (2016) [17]	Yes	Yes	Yes	Yes	Yes	Yes	Yes	Yes	Yes	9 out of 9/good quality
Singer et al. (2003) [32]	Yes	Yes	Yes	Yes	No	Yes	Yes	Yes	Yes	8 out of 9/good quality
Hoeldtke et al. (1998) [37]	Yes	Yes	Yes	Yes	Yes	No	Yes	Yes	Yes	8 out of 9/good quality

Additionally, one cohort study was assessed using the NOS tool [44], as shown in Table 2, and it received an overall score of 8 out of 9.

**Table 2:** Quality appraisal of observational studies using Newcastle-Ottawa Scale (NOS) Selection: maximum of four stars. Comparability: maximum of two stars. Outcome: maximum of three stars. Overall good quality: 7-9 stars.

Study	Study design	Selection	Comparability	Outcome	Total score/overall quality
Axelrod et al. (2005) [36]	Retrospective cohort	****	*	***	8 out of 9/good quality

Study Characteristics

The systematic review analyzed 25 studies, including 20 RCTs, four non-RCTs (NRCTs), and one retrospective cohort study. These studies examined the effectiveness and adverse events (AEs) associated with various drug treatments for NOH. Sample sizes ranged from 6 to 341 participants, highlighting substantial diversity in study design and scope.

The studies covered a broad spectrum of pharmacological agents. Six focused exclusively on droxidopa [14-19], comprising three RCTs and two NRCTs. Midodrine was evaluated in five RCTs [20-24], while atomoxetine was the focus of two RCTs [26,27]. Some studies investigated combination therapies, such as midodrine with pyridostigmine [25] and atomoxetine with pyridostigmine [28], or conducted comparative analyses between drugs like atomoxetine and midodrine [29,30], pyridostigmine and fludrocortisone [33], and fludrocortisone and domperidone [34]. Two studies each examined pyridostigmine [31,32] and fludrocortisone [35,36], while octreotide was evaluated in two studies as well [37,38].

Treatment durations varied among studies. Short-term effects of treatment (<24 hours) were assessed in eight studies [23,28,29,31,32,36-38], involving midodrine, atomoxetine, pyridostigmine, fludrocortisone, and octreotide. In contrast, 17 studies [14-22,24-27,30,33-35] explored long-term effects (>24 hours), with treatment durations ranging from 1 week to 12 months. Long-term evaluations included droxidopa, midodrine, atomoxetine, pyridostigmine, and fludrocortisone. Drug dosages also varied widely: droxidopa (100-600 mg) [14-19], midodrine (2.5-30 mg) [20-25,29,30], atomoxetine (10-18 mg) [26-30], pyridostigmine (30-60 mg) [25,28,31-33], fludrocortisone (0.1-0.2 mg) [33-36], and octreotide (0.5-1.0 µg/kg) [37]. Two studies did not report specific dosage details [24,36].

Most studies measured outcomes based on changes in SBP, DBP, or improvements in symptoms using validated scales, such as the Orthostatic Hypotension Questionnaire (OHQ) [46], Clinical Global Impression (CGI) Scale [47], and Composite Autonomic Symptom Scale (COMPASS-OD). While the head-up tilt (HUT) test remains the gold standard for diagnosing OH [1], achieving meaningful symptom improvement is often the more clinically significant goal. The effectiveness of treatments is typically measured through established tools such as the OHQ [46], the CGI [47] rating scale, and the Global Symptom Relief Score [20,22]. The OHQ [46], developed by Kaufmann et al. in 2012, assesses changes in symptom burden among OH patients through two components: the Orthostatic Hypotension Symptom Assessment (OHSA) and the Orthostatic Hypotension Daily Activity Scale (OHDAS). The CGI includes complementary scales for assessing symptom severity and treatment progress [47]. AEs were reported in nearly all studies, except for two [29,31]. Detailed findings, including outcomes and reported AEs, are presented in Table 3.

**Table 3:** Summary of study characteristics AE: adverse event, AN: autonomic neuropathy, CGI: Clinical Global Impression, CGI-S: Clinical Global Impression of Severity, COMPASS-OD: Composite Autonomic Symptom Scale, DBH: dopamine-β-hydroxylase, DBP: diastolic blood pressure, MSA: multiple system atrophy, NRCT: non-randomized controlled trial, OH: orthostatic hypotension, OHDAS: Orthostatic Hypotension Daily Activity Scale, OHQ: Orthostatic Hypotension Questionnaire, OHSA: Orthostatic Hypotension Symptom Assessment, PAF: pure autonomic failure, PD: Parkinson's disease, RCT: randomized controlled trial, SBP: systolic blood pressure. *p > 0.05 (values not significant).

Author/year	Study design	Sample size	Population characteristics	Intervention/control	Duration of treatment/follow-up	Outcome (mean) (+, increase; -, decrease)	Adverse effects reported
Hauser et al. (2022) [14]	Open-label NRCT	114	Sex: 72 M, 42 F. Mean age: 68 ± 13.4 years. Diagnosis: PD (52%), nondiabetic AN (30%), PAF (14%), MSA (4%)	Droxidopa (100-600 mg 3 times daily)	12 weeks of open-label treatment	Change in OHSA composite score: -3.3 ± 1.9. Change in OHDAS composite score: -3.4 ± 2.4. Increase in supine SBP: 15.5 ± 22.9 mmHg	Droxidopa: overall, 64% (falls, 17%; headache, 13%; dizziness, 9%; nausea, 7%)
Kaufmann et al. (2014) [15]	Parallel group, placebo-controlled RCT	168	Sex: 84 M, 78 F. Mean age: 56.6 ± 16.9 years. Diagnosis: PD (40.7%), PAF (33.3%), MSA (16.0%), nondiabetic AN (4.9%), other (4.9%)	Droxidopa (100-600 mg 3 times daily) vs. placebo	2-week open-label dose titration, 1-week washout, 1-week randomized study period	Change in OHQ composite score: droxidopa (-1.83 ± 2.07 units) vs. placebo (-0.93 ± 1.69 units) (difference: 0.90 units, favoring droxidopa). Change in OHSA composite score: droxidopa (-1.68 ± 2.13 units) vs. placebo (-0.95 ± 1.90 units) (difference: 0.73 units, favoring droxidopa). Change in OHDAS composite score: droxidopa (-1.98 ± 2.31 units) vs. placebo (-0.92 ± 1.82 units) (difference: 1.06 units, favoring droxidopa). Increase in standing SBP: droxidopa (+11.2 mmHg) vs. placebo (+3.9 mmHg) (difference: +7.3 mmHg)	Droxidopa: overall, 18.5% (headache, 7.4%; dizziness, 3.7%). Placebo: overall, 14.8% (dizziness, 1.2%; falls, 3.7%)
Biaggioni et al. (2015) [16]	Parallel group, placebo-controlled RCT	101	Sex: 62 M, 39 F. Mean age: 64.9 ± 12.6 years. Diagnosis: PD (43.6%), PAF (17.8%), MSA (29.7%), nondiabetic AN (5.0%), DBH deficiency (1.0%), other (3.0%)	Droxidopa (100-600 mg 3 times daily) vs. placebo	2-week open-label dose titration, 2-week randomized study period	Change in OHQ composite score: droxidopa (+0.11 ± 2.18 units) vs. placebo (+1.22 ± 2.39 units). Change in OHSA item 1 (dizziness/lightheadedness) score: droxidopa (1.3* ± 2.8 units) vs. placebo (1.9 ± 3.2 units). Change in standing SBP: droxidopa (-7.6* ± 19.7 mmHg) vs. placebo (-5.2 ± 26.8 mmHg)	Droxidopa: overall, 30% (falls, 2%; headache, 4%; dizziness, 4%. Placebo: overall, 37.3% (falls, 11.8%; headache, 7.8%; dizziness, 2%)
Isaacson et al. (2016) [17]	Open-label NRCT	102	Sex: 61 M, 42 F. Mean age: 65.8 ± 12.3 years. Diagnosis: PD (50.7%), PAF (20.0%), MSA (22.7%), nondiabetic AN (2.7%), DBH deficiency (1.3%), other (2.7%)	Droxidopa (100-600 mg 3 times daily)	12 months of open-label treatment	Change in OHQ composite score: -3.29. Increase in standing SBP: 12.3 ± 26.6 mmHg. CGI-S clinician ratings: baseline marked OH (67.6%) vs. 12-month marked OH (17.8%). CGI-S patient ratings: baseline marked OH (67.6%) vs. 12-month marked OH (12.2)	Droxidopa: falls (20.6%), urinary tract infections (17.6%), headache (13.7%), syncope (12.7%), back pain (10.8%), and dizziness (7.8%)
Hauser et al. (2014) [18]	Parallel group, placebo-controlled RCT	51	Sex: 31 M, 20 F. Mean age: 72.6 ± 7.6 years. Diagnosis: PD (100%)	Droxidopa (100-600 mg 3 times daily) vs. placebo	2-week double-blind dose titration, 8-week randomized study period	Change in OHQ composite score: droxidopa (-2.2* ± 2.4 units) vs. placebo (-2.1 ± 2.5 units). Change in OHSA item 1 (dizziness/lightheadedness) score: droxidopa (-2.3 ± 3.0 units) vs. placebo (-1.0 ± 3.0 units). Increase in standing SBP: droxidopa (+7.0 mmHg) vs. placebo (+7.7 mmHg). Number of total falls: droxidopa (72) vs. placebo (192)	Droxidopa: 71% of participants reported at least one AE. Placebo: 85% of participants reported at least one AE
Hauser et al. (2015) [19]	Parallel group, placebo-controlled RCT	171	Sex: 97 M, 50 F. Mean age: 72.2 ± 7.84 years. Diagnosis: PD (100%)	Droxidopa (100-600 mg 3 times daily) vs. placebo	2-week dose titration, 8-week randomized study period	Change in OHSA item 1 (dizziness/lightheadedness) score at week 1: droxidopa (-2.3 ± 2.95 units) vs. placebo (-1.3 ± 3.16 units). Increase in standing SBP at week 1: droxidopa (+6.4 ± 18.85 mmHg) vs. placebo (+0.7 ± 20.18 mmHg). Change in OHSA item 1 (dizziness/lightheadedness). Score at week 8: droxidopa (-2.1* ± 3.03 units) vs. placebo (1.5 ± 2.91 units). Change in OHQ composite score: droxidopa (-2.2* ± 2.9 units) vs. placebo: (-2.0 ± 2.18 units)	Droxidopa: overall, 82% (headache, 13.5%; dizziness, 10.1%; nausea, 7.9%; hypertension, 7.9%). Placebo: overall, 79.3% (headache, 7.3%; dizziness, 4.9%; nausea, 2.4%; hypertension, 4.9%)
Low et al., 1997 [20]	Parallel group, placebo controlled RCT	171	Sex: 81 M, 81 F. Mean age: 59.48 ± 15.69 years. Diagnosis: Shy-Drager syndrome (25%), Bradbury-Eggleston syndrome (23%), diabetes mellitus (23%), other (18%), PD (12%)	Midodrine (10 mg 3 times daily) vs. placebo	1-week single-blind placebo run-in period, 3-week double-blind treatment phase, 2-week single-blind placebo washout period	Increase in standing SBP compared to placebo: 22.4 mmHg. Global symptom relief scores by investigator: midodrine (2.8 ± 0.2) vs. placebo (2.0 ± 0.1). Global symptom relief scores by patients: midodrine (2.7 ± 0.2) vs. placebo (2.2 ± 0.1)	Midodrine: pilomotor reactions (13%), pruritus, especially scalp (10%), paresthesia (9%), urinary retention (6%), supine hypertension (4%). Placebo: pruritus, especially scalp (2%), paresthesia (3%)
Jankovic et al. (1993) [21]	Parallel group, placebo-controlled RCT	97	Sex: 53 M, 44 F. Mean age: 61 years. Diagnosis: diabetes mellitus (50.9%), PD (41.5%), Bradbury-Eggleston syndrome (37.7%), Shy-Drager syndrome (34.0%), other (18.9%)	Midodrine (2.5 mg or 5 mg or 10 mg, 3 times daily) vs. placebo	1-week single-blind placebo run-in period, 4-week double-blind treatment phase	Increase in standing SBP, 1-hour post-dose: 2.5 mg midodrine (9* mmHg), 5 mg midodrine (4* mmHg), 10 mg midodrine (22 mmHg) vs. placebo (3 mmHg). Symptom improvement (percentage improvement from baseline). (1) Dizziness/lightheadedness: 2.5 mg midodrine (39%*), 5 mg midodrine (79%), 10 mg midodrine (69%) vs. placebo (14%). (2) Weakness/fatigue: 2.5 mg midodrine (44%*), 5 mg midodrine (50%*), 10 mg midodrine (44%*) vs. placebo (14%). (3) Blurred vision: 2.5 mg midodrine (39%*), 5 mg midodrine (63%), 10 mg midodrine (56%) vs. placebo (-5%, worsened). (4) Syncope (fainting/falling): 2.5 mg midodrine (33%), 5 mg midodrine (33%), 10 mg midodrine (54%) vs. placebo (21%). (5) Energy level: 2.5 mg midodrine (78%), 5 mg midodrine (67%), 10 mg midodrine (119%) vs. placebo (29%). (6) Standing time (>15 minutes): 2.5 mg midodrine (61%), 5 mg midodrine (26%*), 10 mg midodrine (32%*) vs. placebo (0%). (7) Depression: 2.5 mg midodrine (11%*), 5 mg midodrine (22%), 10 mg midodrine (23%) vs. placebo (0%)	Midodrine: overall, 27% (scalp pruritus/tingling, 13.5%; supine hypertension, 8%; urinary urgency, 4%; headache, 3%. Placebo: overall, 22% (scalp pruritus/tingling, 2%; supine hypertension, 1%; headache, 1%)
Wright et al. (1998) [22]	Crossover group, placebo-controlled RCT	27	Sex: 11M, 14 F. Mean age: 62 years. Diagnosis: PAF (Bradbury-Eggleston syndrome) (56.0%), MSA (Shy-Drager syndrome) (28.0%), diabetic AN (12.0%), PD (4.0%)	Midodrine (2.5 mg or 10 mg or 20 mg, single dose) vs. placebo	6-day study duration: days 1 and 6, no medication; days 2-5, randomized single-dose treatments (placebo or midodrine) with 24-hour washout between doses	Increase in standing SBP, 1-hour post-dose: 2.5 mg midodrine (7* mmHg), 109 mg midodrine (34 mmHg), 20 mg midodrine (43 mmHg) vs. placebo (5 mmHg). Global symptom relief scores by investigator: 2.5 mg midodrine (2.3* ± 0.2), 10 mg midodrine (3.3 ± 0.2), 20 mg midodrine (3.3 ± 0.2) vs. Placebo (1.9 ± 0.2). Global symptom relief scores by patients: 2.5 mg midodrine (2.7* ± 0.2), 10 mg midodrine (3.3 ± 0.2), 20 mg midodrine (3.3 ± 0.2) vs. placebo (2.0 ± 0.2)	Midodrine: overall, 60% (15/25 cases) (pilomotor reactions (goosebumps, tingling, pruritus): 2.5 mg midodrine, 4 cases; 10 mg midodrine, 11 cases; 20 mg midodrine, 11 cases; hypertension: 10 mg midodrine, 17%; 20 mg midodrine, 41%; severe reaction: 20 mg midodrine, 2 cases)
ClinicalTrials.gov (ID NCT00555880) [23]	Crossover group, placebo-controlled RCT	24	Sex: 12 M, 12 F. Mean age: 60 ± 15.46 years. Diagnosis: neurogenic orthostatic hypotension (100%)	Midodrine (10-30 mg, one dose) vs. placebo	Each treatment lasted one day, with one-day washout period in between	OHSA composite scores (1-hour post-treatment): midodrine (13.7* ± 2.95 units) vs. placebo (19.4* ± 2.95 units). SBP (1-hour post-dose): midodrine (106.5 ± 37.27 mmHg) vs. placebo (90.2 ± 24.17 mmHg)	Midodrine: 23.08% (cardiac disorder, nausea, vomiting, dizziness, headache). Placebo: 18.18% (hypertension)
ClinicalTrials.gov (ID NCT00046475) [24]	Crossover group, placebo-controlled RCT	104	Sex: 57 M, 47 F. Mean age: 62.2 ± 14.27. Diagnosis: neurogenic orthostatic hypotension (100%)	Midodrine vs. placebo	2-week washout period, 1-week titration period, 2-week treatment period, 2-week crossover treatment period	Change in OHSA composite score: midodrine (-1.3 ± 2.5 units) vs. placebo (-0.54 ± 1.95 units). Change in OHDAS composite score: midodrine (-1.4 ± 2.6 units) vs. placebo (-0.4 ± 1.9 units). Increase in standing SBP: midodrine (10.7 mmHg) vs. placebo (2.8 mmHg).Change in supine SBP: midodrine (7.6 mmHg) vs. placebo (-0.9 mmHg)	Midodrine: 21.15% (pruritus, 7.69%; headache, 5.77%; paresthesia, 4.81%; dizziness, 2.88%; hypertension, 2.88; UTI, 0.96). Placebo: 21.90% (pruritus, 7.62%; headache, 6.67%; paresthesia, 4.76%; dizziness, 4.76%; hypertension, 1.9; UTI, 0.95)
Byun et al. (2017) [25]	Parallel group, RCT	87	Sex: 41 M, 46 F. Mean age: 57.2 ± 16 years. Diagnosis: idiopathic OH (47.1%), nondiabetic AN (25.3%), diabetic AN (23.0%), MSA (4.6%)	Midodrine only: 2.5-5 mg, twice daily. Pyridostigmine only: 30-60 mg, twice daily. Combination: 2.5 mg midodrine + 30 mg pyridostigmine, twice daily	3 months	Change in OHQ composite score: midodrine (-19.3 units) vs. pyridostigmine (-14.6 units) vs. combination (-15.5 units). Change in OHSA composite score: midodrine (-10.5 units) vs. pyridostigmine (-9.2 units) vs. combination (-9.7 units). Change in OHDAS composite score: midodrine (-8.9 units) vs. pyridostigmine (-5.4 units) vs. combination (-5.8 units). Drop in orthostatic SBP: midodrine (-12.2* ± 12.8 mmHg) vs. pyridostigmine (-11.7* ± 14.7 mmHg) vs. combination (11.9* ± 12.0 mmHg)	Midodrine only: one patient (4.3%) (headache, aggravated dizziness). Pyridostigmine only: six patients (25.0%) (aggravated dizziness, 5 patients; headache, 2 patients; gastrointestinal symptoms, 2 patients; limb tremors, 1 patient). Combination group: three patients (11.5%) (abdominal pain, nausea, 2 patients; dizziness, 1 patient; visual disturbances, 1 patient)
Mwesigwa et al. (2024) [26]	Crossover group, placebo-controlled RCT	40	Sex: 24 M, 16 F. Mean age: 67.9 ± 8.1 years. Diagnosis: MSA (41%), PAF (41%), PD (19%)	Atomoxetine (10 mg or 18 mg twice daily) vs. placebo	4-week randomized phase, 1-week washout period, 4-week crossover randomized phase	Change in OHQ composite score: atomoxetine (-1.0* ± 1.4 units) vs. placebo (-1.0* ± 1.5 units). Change in OHSA composite score: atomoxetine (-1.0* ± 1.0 units) vs. placebo (-0.8* ± 1.2 units). Change in OHDAS composite score: atomoxetine (-1.4* ± 1.8 units) vs. placebo (-1.6* ± 1.6 units). Change in standing SBP: week 2, atomoxetine (+11.0 ± 15.5 mmHg) vs. placebo (-0.4 ± 13.9 mmHg); week 4, atomoxetine (+11* ± 18.1 mmHg) vs. placebo (+3* ± 14.7 mmHg)	Atomoxetine: 28 events reported. Placebo: 20 events reported
ClinicalTrials.gov (ID NCT02784535) [27]	Crossover group, placebo-controlled RCT	40	Sex: 16 M, 24 F. Mean age: 67.9 ± 8.1 years. Diagnosis: PD or PAF (55.0%), MSA (45.0%).	Atomoxetine (10 mg or 18 mg, 3 times daily) vs. placebo	4-week randomized phase, 1-week washout period, 4-week crossover randomized phase	Change in OHQ composite score: atomoxetine (-0.5 ± 1.58 units) vs. placebo (-0.58 ± 2.39 units). Change in standing SBP: atomoxetine (-2.44 mmHg) vs. placebo (3.88 mmHg). Change in standing DBP: atomoxetine (-0.94 mmHg) vs. placebo (-3.06 mmHg)	Atomoxetine: 32.5% (reflux esophagitis, 5%; altered sensation, 12.5%; UTI, 7.5%; upper respiratory infections, 12.5%; fall injury, 2.5%; headache, 2.5%). Placebo: 32.5% (reflux esophagitis, 2.5%; altered sensation, 7.5%; altered bowel movement, 5%; upper respiratory infections, 2.5%; fall injury, 5%; headache, 7.5%)
Okamoto et al. (2019) [28]	Crossover group, placebo-controlled RCT	12	Sex: 7 M, 5 F. Mean age: 69 ± 3 years. Diagnosis: PAF (41.7%), MSA (25.0%), PD (16.7%), amyloidosis (8.3%), autonomic failure of unknown cause (8.3%)	Atomoxetine: 18 mg single oral dose. Pyridostigmine: 60 mg single oral dose. Combination: atomoxetine (18 mg) + pyridostigmine (60 mg) (single oral dose) vs. placebo	Each treatment administered on separate days, with at least one day in between	Seated SBP at 60-minute post-drug: atomoxetine (105* ± 5 mmHg), pyridostigmine (99* ± 6 mmHg), combination (133 ± 9 mmHg) vs. placebo (107* ± 6 mmHg). Change in standing SBP (1-minute post-drug): atomoxetine (+14* ± 6 mmHg), pyridostigmine (-1* ± 5 mmHg), combination (+20 ± 9 mmHg) vs. placebo (-2* ± 4 mmHg). OHSA composite scores at 60-minute post-drug: atomoxetine (28.0* ± 6.8), pyridostigmine (29.6* ± 5.6), combination (19.9 ± 5.0) vs. placebo (29.2 ± 4.5)	No serious adverse events noted. Combination treatment was considered a safe and well-tolerated profile for most patients
Ramirez et al. (2014) [29]	Crossover group, placebo-controlled RCT	69	Sex: 38 M, 31 F. Mean age: 65 ± 9 years. Diagnosis: PAF (37.7%), MSA (30.4%), PD (17.4%).	Atomoxetine (18 mg), midodrine (5-10 mg) vs. placebo	Each treatment administered in separate sessions	Change in standing SBP: atomoxetine vs. placebo (+20 mmHg), midodrine vs. placebo (+12 mmHg), atomoxetine vs. midodrine (+7.5 mmHg). Change in seated SBP: atomoxetine vs. placebo (+20 mmHg), midodrine vs. placebo (+20 mmHg), atomoxetine vs. midodrine (0.3* mmHg). Change in OHQ composite score: atomoxetine vs placebo (0.4 units), midodrine vs. placebo (-0.5* units), atomoxetine vs. midodrine (no difference). Change in OHSA item 1 (dizziness/lightheadedness) score: atomoxetine vs. placebo (-0.6 units), midodrine vs. placebo (-0.6* units), atomoxetine vs. midodrine (no difference)	Not specified
Byun et al. (2020) [30]	Parallel group, RCT	50	Sex: 28 M, 22 F. Mean age: 63.1 ± 9.6 years. Diagnosis: idiopathic neurogenic OH (58.0%), MSA (16.0%), diabetic AN (16.0%), or nondiabetic AN (10.0%)	Atomoxetine (18 mg daily) vs. midodrine (5 mg twice daily)	3 months	Orthostatic drop in SBP at 1 month: atomoxetine (12.3 ± 12.5 mmHg) vs. baseline (25.8 ± 11.7), midodrine (16.2 ± 16.7 mmHg) vs. baseline (26.7 ± 10.9). OHQ composite score at 1 month: atomoxetine (35 ± 20.1) vs. baseline (44.7 ± 23.9), midodrine (33.4* ± 23.4) vs. baseline (37.5 ± 33.4). OHSA composite score at 1 month: atomoxetine (21.0 ± 13.6) vs. baseline (26.5 ± 15.7), midodrine (19.1* ± 13.2) vs. baseline (22.1 ± 12.4). OHDAS composite score at 1 month: atomoxetine (14.0 ± 8.3) vs. baseline (18.2 ± 11.2), midodrine (14.3* ± 11.1) vs. baseline (15.4 ± 10.7)	Atomoxetine: 13% (3/23 cases) (frequent sweating, 2 cases), frequent urination, 1 case). Midodrine: 10.5% (2/19 cases) (worsening dizziness, 1 case; agitation, 1 case)
Singer et al. (2006) [31]	Crossover group, placebo-controlled RCT	58	Sex: 30 M, 28 F. Mean age: 59 ± 11 years. Diagnosis: MSA (29.3%), PAF (25.9%), diabetic AN (19.0%), autoimmune AN (15.5%), unspecified neurogenic OH (10.3%)	Pyridostigmine bromide(60 mg), pyridostigmine bromide (60 mg) + midodrine hydrochloride (2.5 mg), pyridostigmine bromide (60 mg) + midodrine hydrochloride (5 mg) vs. placebo	Each treatment phase lasted one day, with follow-up monitoring for 6 hours post-treatment	Change in standing SBP (6-hour post-treatment): pyridostigmine (+10* mmHg), pyridostigmine + 2.5 mg midodrine (+12* mmHg), pyridostigmine + 5.0 mg midodrine (+15* mmHg), placebo (+8* mmHg). Fall in standing DBP (1-hour post-treatment): pyridostigmine (27.6 mmHg), pyridostigmine + 2.5 mg midodrine (27.2 mmHg), pyridostigmine + 5.0 mg midodrine (27.2 mmHg), placebo (34.0 mmHg)	Not specified
Singer et al. (2003) [32]	Open-label NRCT	15	Sex: 8 M, 7 F. Mean age: 56.2 ± 4.8. Diagnosis: MSA (46.7%), PD (20.0%), idiopathic AN (20.0%), diabetic AN (6.7%), or amyloidosis (6.7%)	Pyridostigmine: 60 mg, single dose	One-hour post-treatment assessment	Standing SBP (1-hour post-treatment): baseline (110.9 mmHg) vs. after (124.3 mmHg). Standing DBP (1-hour post-treatment): baseline (58.7 mmHg) vs. after (66.5 mmHg). Supine SBP (1-hour post-treatment): baseline (155.5 mmHg) vs. after (158.0* mmHg). Supine DBP (1-hour post-treatment): baseline (70.4 mmHg) vs. after (74.5* mmHg). Significant improvement in orthostatic symptoms, comparing the symptoms before and after treatment using Wilcoxon rank-sum test	Sweating and perspiration (1 case), urinary urgency (1 case), abdominal cramping (1 case)
Schreglmann et al. (2017) [33]	Crossover group, RCT	13	Sex: 11 M, 2 F. Mean age: 71.3 ± 5.6 years. Diagnosis: PD (100%)	Pyridostigmine bromide (60 mg, thrice daily) vs. fludrocortisone (0.2 mg, once daily)	2-week treatment phase, 3-week washout period, 2-week crossover treatment period	Supine SBP: pyridostigmine (130.4 ± 18.3 mmHg), fludrocortisone (143.2 ± 10.1 mmHg) vs. baseline (128.4 ± 12.8 mmHg) (favoring fludrocortisone). Standing SBP: pyridostigmine (100.2 ± 18.8 mmHg), fludrocortisone (112.0 ± 21.8 mmHg) vs. baseline (105.0 ± 16.8 mmHg). Orthostatic drop in SBP on Schellong manoeuver: pyridostigmine (36.4* ± 11.1 mmHg), fludrocortisone (34.6* ± 15.2 mmHg) vs. baseline (41.1* ± 16.9 mmHg). Orthostatic drop in DBP on Schellong manoeuver: pyridostigmine (22.1 ± 17.0 mmHg), fludrocortisone (14.0 ± 12.6 mmHg) vs. baseline (22.9 ± 13.6 mmHg). OHSA composite score: pyridostigmine (16.0* ± 10.8), fludrocortisone (16.6* ± 14.3) vs. baseline (17.8* ± 8.4). OHDAS composite score: pyridostigmine (10.9* ± 5.0), fludrocortisone (12.4* ± 10.6) vs. baseline (11.5* ± 6.6)	Pyridostigmine bromide: 3 cases (dizziness, 1 case; dry mouth, 1 case; PD increase of motor “OFF” phase, 1 case). Fludrocortisone: 1 case (Mild leg edema)
Schoffer et al. (2007) [34]	Crossover group, RCT	13	Sex: 12 M, 5 F. Mean age: 69 ± 11 years. Diagnosis: PD (100%).	Phase II: fludrocortisone (0.1 mg), once daily in the morning + placebo tablets at lunch and supper vs. domperidone (10 mg), three times daily	Phase II: 3-week treatment phase, 1-week washout period, 3-week crossover treatment period	COMPASS-OD score: fludrocortisone (6 ± 3 units), domperidone (6 ± 2 units) vs. baseline (9 ± 3 units). Average CGI score improved to: fludrocortisone (1 ± 1.2 units), domperidone (1 ± 1.2 units). Orthostatic drop in SBP: fludrocortisone (24* ± 23 mmHg), domperidone (19* ± 21 mmHg) vs. baseline (32* ± 23 mmHg). Orthostatic drop in DBP: fludrocortisone (20* ± 12 mmHg), domperidone (6* ± 15 mmHg) vs. baseline: (20* ± 10 mmHg)	Fludrocortisone: 6 cases (nausea, 2 cases; chest pain, 1 case; morning headache, 1 case; lightheadedness, 1 case; dizziness, 1 case). Domperidone: 5 cases (nausea, 2 cases; chest pain, 1 case; abdominal pain, 1 case; palpitations, 1 case; headache, 1 case)
Campbell et al. (1975) [35]	Crossover group, placebo-controlled RCT	6	Sex: 6 M, 0 F. Age: 33-64 years. Diagnosis: diabetic AN (100%).	Fludrocortisone acetate (0.1 mg), twice daily vs. placebo	3-week treatment phase, 3-week washout period, 3-week crossover treatment period	Supine SBP: fludrocortisone (180 ± 26 mmHg) vs. placebo (149 ± 21 mmHg). Tilted SBP: fludrocortisone (154 ± 29 mmHg) vs. placebo (110 ± 16 mmHg). Tilted DBP: fludrocortisone (88 ± 11 mmHg) vs. placebo (76 ± 4 mmHg). Four out of five participants reported marked improvement during fludrocortisone	Participants with low baseline serum albumin developed pitting ankle edema within a week of starting fludrocortisone. One participant reported frontal headache and breathlessness
Axelrod et al. (2005) [36]	Retrospective observational study	341	Sex: 163 M, 178 F. Age: 24-82 years. Diagnosis: familial dysautonomia (100%)	Fludrocortisone vs. placebo	Fludrocortisone use was analyzed from initiation in 1976 with up to 29 years of data	Increase in mean blood pressure post-treatment (vs. pre-treatment): supine 5 minute, increased by 19 mmHg; erect 1 minute, increased by 15 mmHg; erect 5 minutes, increased by 13 mmHg. Orthostatic symptoms: dizziness (before treatment, 78.7%; after treatment, 32.3%), leg cramps (before treatment, 37.1%; after treatment, 11.9%), syncope (before treatment, 34.6%*; after treatment, 13.2%*), headache (before treatment, 37.7%*; after treatment, 18.5%*)	Fludrocortisone: overall, 9% (16/175 patients) (hypertension, 5 cases; dizziness, 5 cases; weakness, 3 cases; headache, 2 cases; edema, 2 cases; nausea, 1 cases)
Hoeldtke et al. (1998) [37]	NRCT	16	Sex: NA. Age: 50-83 years. Diagnosis: PAF (62.5%), diabetic AN (25.0%), MSA (6.3%), chronic renal failure (6.3%)	Protocol 3: midodrine (5 mg or 10 mg orally) and/or octreotide (0.5-1.0 µg/kg subcutaneously) or combination (midodrine + octreotide) vs. placebo	Each treatment administered in separate sessions	Standing mean BP. Midodrine (10 mg): modest improvement in standing BP compared to no treatment. Octreotide (1.0 µg/kg): improved BP significantly during standing. Combination therapy (midodrine + octreotide): synergistic improvement in standing BP compared to either drug alone. Placebo: blood pressure dropped significantly during standing. Mean standing time (minutes before symptoms or hypotension): midodrine (10 mg), 8.4* ± 2.7 minutes; octreotide (1.0 µg/kg), 13.2 ± 3.9 minutes; combination therapy (midodrine + octreotide), 21.2 ± 5.5 minutes; no treatment, 3.5 ± 0.7 minutes	Midodrine: pruritus of the scalp (6 cases), urinary urgency (2 cases), supine hypertension (3 patients). Octreotide: nausea/loose stools (8 patients)
Bordet et al. (1995) [38]	Crossover group, placebo-controlled RCT	9	Sex: 3 M, 6 F. Age: 61-83 years. Diagnosis: MSA (100%).	Octreotide (100 µg) subcutaneously, once vs. placebo	30-minute post-treatment assessment. Test repeated 3 consecutive days	Supine SBP: octreotide (175 ± 9 mmHg), placebo (150 ± 8 mmHg), control (153 ± 10 mmHg). Supine DBP: octreotide (96 ± 4.3 mmHg), placebo (80.5 ± 4 mmHg), control (83 ± 5 mmHg)	Well-tolerated, no adverse events reported

Droxidopa: Efficacy and Safety Profile

Droxidopa, a prodrug of norepinephrine, plays a crucial role in the management of OH by enhancing peripheral vasoconstriction, which leads to improved standing blood pressure [15]. This medication is typically administered three times daily, with dosages ranging from 100 mg to 600 mg [14-19]. Numerous studies have highlighted its effectiveness in alleviating OH symptoms, with reported increases in standing systolic blood pressure ranging from approximately 7 to 12 mmHg.

For instance, Kaufmann et al. [15] demonstrated that patients receiving droxidopa experienced a decrease in the OHQ composite score of 1.83, compared to a reduction of only 0.93 in the placebo group. Additionally, there was a notable increase in standing SBP of 11.2 mmHg for those on droxidopa, contrasted with just 3.9 mmHg for the placebo group. Similarly, Biaggioni et al. [16] observed significant improvements in the OHQ composite score in a two-week RCT, further reinforcing the drug's efficacy. However, variability in treatment responses has been noted, as two RCTs indicated no significant results after eight weeks of treatment [18,19]. This highlights the need for individualized patient assessment and monitoring.

In terms of safety, the most commonly reported adverse effects associated with droxidopa include headache, dizziness, and nausea [14-19].

Notably, urinary tract infections were reported in approximately 17% of patients undergoing treatment for 12 months. Despite these potential side effects, one of the key advantages of droxidopa over midodrine is its minimal incidence of supine hypertension, which can be a significant concern with other treatments.

In a 12-week open-label study conducted by Hauser et al. [14], significant reductions in the OHSA and OHDAS composite scores were documented, with decreases of 3.3 and 3.4, respectively, compared to baseline. Another study [17] assessing droxidopa over a 12-month period demonstrated clinically significant symptom improvement, evidenced by a 3.29 decrease in the OHQ composite score and enhanced CGI-S ratings from both clinicians and patients.

While droxidopa is effective in managing OH symptoms and improving blood pressure, its safety profile and relative advantages over midodrine, particularly regarding supine hypertension, make it a valuable option in the pharmacological arsenal against this condition.

Midodrine: Efficacy and Safety Profile

Midodrine, an alpha-adrenergic agonist, is utilized in the treatment of OH due to its vasoconstrictive properties that effectively elevate blood pressure [20]. It is administered orally three times daily, with total daily doses ranging from 2.5 mg to a maximum of 30 mg [20-22]. Numerous studies have substantiated its efficacy, with notable research conducted by Low et al. [20] and Jankovic et al. [21], demonstrating significant improvements in standing SBP. In these studies, increases of up to 22 mmHg were observed, particularly at higher dosages, along with a reduction in symptoms such as dizziness and syncope within a treatment period of 3-4 weeks.

Further supporting the drug's effectiveness, one clinical trial reported a decrease in the OHSA composite score of 1.3 with midodrine, compared to a mere 0.54 reduction in the placebo group. Additionally, the trial noted an increase in standing SBP of 10.7 mmHg for the midodrine group versus 2.8 mmHg for the placebo after just two weeks of treatment [24]. Moreover, two trials assessing the efficacy of midodrine as a single dose revealed significant improvements in standing SBP and global symptom relief scores, particularly with doses exceeding 10 mg. Notably, a combination therapy involving 2.5 mg of midodrine and 30 mg of pyridostigmine, as evaluated by Byun et al. [25], demonstrated enhanced orthostatic SBP compared to either medication alone. However, it is important to note that the improvement in symptoms was found to be greater with midodrine alone.

Midodrine provides notable therapeutic benefits but is associated with certain side effects. Commonly reported adverse effects include pruritus, particularly on the scalp, urinary retention, and an increased risk of supine hypertension, which can be managed by avoiding nighttime dosing [21,22]. Minor side effects, such as pilomotor reactions, nausea, and headache, have also been documented and are generally well-tolerated [21-24].

Atomoxetine: Efficacy and Safety Profile

Atomoxetine, primarily recognized as a norepinephrine reuptake inhibitor, has garnered attention as a promising treatment for NOH, particularly among patients with autonomic failure [28]. By increasing synaptic concentrations of norepinephrine, atomoxetine aids in improving SBP and alleviating associated symptoms [26].

Clinical studies have indicated that the effects of atomoxetine can be comparable to those of midodrine, another medication commonly used for this condition. For instance, Ramirez et al. [29] reported a substantial 20 mmHg increase in standing SBP with atomoxetine, in contrast to a 12 mmHg increase observed with midodrine when both were compared to placebo. Additionally, atomoxetine significantly improved the OHQ composite score, highlighting its potential benefits in enhancing patient-reported outcomes. In a subsequent study by Byun et al. [30], atomoxetine exhibited significant improvements in symptom scores after one month of treatment compared to midodrine.

Despite these promising findings, two crossover RCTs [26,27] evaluating atomoxetine at daily doses of 10 mg or 18 mg for four weeks did not demonstrate significant improvements over placebo. However, a smaller study conducted by Okamoto et al. [28] involving 12 participants explored the hypothesis that combining pyridostigmine with atomoxetine could enhance the pressor effects of atomoxetine, thereby improving orthostatic tolerance and symptoms in patients with severe autonomic failure. The results indicated that this combination therapy led to statistically significant improvements in both standing SBP and the OHSA composite score.

While the efficacy of atomoxetine is well-documented, its clinical application may be constrained by individual tolerability. Reported side effects include altered sensations, urinary tract infections, and upper respiratory symptoms, which can affect patient adherence to treatment [27,30].

Pyridostigmine: Efficacy and Safety Profile

Pyridostigmine, an acetylcholinesterase inhibitor, has been explored as a treatment option for NOH, providing modest benefits in managing this condition [31]. Clinical studies have indicated slight improvements in standing blood pressure and symptom relief, particularly when pyridostigmine is used in combination with midodrine [25].

In a study conducted by Singer et al. [31], the efficacy of pyridostigmine was compared both as a standalone treatment and in conjunction with midodrine. The findings revealed a significant improvement in diastolic blood pressure, which is likely attributable to increased total peripheral resistance resulting from the drug's action. Furthermore, an open-label study [32] involving a dosage of 60 mg of pyridostigmine demonstrated an increase in standing SBP from a baseline of 110.9 mmHg to 124.3 mmHg, underscoring its potential effectiveness in enhancing blood pressure levels.

Despite these positive outcomes, the use of pyridostigmine is not without its challenges. The elevation of acetylcholine levels associated with this medication can precipitate a range of adverse effects, including sweating, urinary urgency, dizziness, and gastrointestinal disturbances [32]. These side effects can significantly impact a patient's quality of life and may limit the drug's utility as a monotherapy for NOH.

Fludrocortisone: Efficacy and Safety Profile

Fludrocortisone, a mineralocorticoid, is widely utilized to expand plasma volume and increase blood pressure in patients with NOH [35]. The review of the literature reveals significant improvements in both supine and standing BP, with some studies reporting increases of up to 30 mmHg [35].

A notable study by Schreglmann et al. [33] assessed 13 participants with PD and found a 37% reduction in DBP drop during orthostatic challenges, alongside an 11% increase in peripheral supine SBP with fludrocortisone. These results suggest that fludrocortisone may exhibit superior efficacy compared to pyridostigmine in managing OH.

Two crossover RCTs, albeit with small sample sizes, evaluated the efficacy of a daily dosage of 0.1 mg of fludrocortisone over three weeks. These studies demonstrated comparable symptom improvement to domperidone [34] and a significant increase in supine SBP compared to placebo [35]. Additionally, fludrocortisone has shown particular effectiveness for chronic management, as evidenced by a retrospective cohort study conducted by Axelrod et al. [36]. This study included 341 participants and reported statistically significant improvements in mean blood pressure and reductions in OH symptoms, such as a 46% decrease in dizziness and a 25% decrease in leg cramps.

Despite its therapeutic benefits, fludrocortisone is associated with notable side effects, including leg edema, headaches, hypokalemia and supine hypertension [34-36]. These risks are particularly pronounced in individuals with low baseline serum albumin levels [35], necessitating careful patient selection and monitoring. While long-term observational data affirm its efficacy, regular follow-up is essential to mitigate potential adverse effects.

Octreotide: Efficacy and Safety Profile

Octreotide, a somatostatin analog, plays a role in managing NOH by reducing splanchnic blood pooling. This mechanism enhances effective circulating blood volume, thereby improving blood pressure and alleviating symptoms of NOH, particularly in short-term studies [38].

Research has demonstrated the effectiveness of octreotide in combination with other medications. For instance, a study by Hoeldtke et al. [37] showed that combining 10 mg of oral midodrine with 1.0 µg/kg of subcutaneous octreotide resulted in greater improvements in mean standing blood pressure and delayed the onset of OH symptoms compared to either drug used alone [37]. Additionally, a crossover RCT by Bordet et al. [38] highlighted octreotide's efficacy, reporting an increase in supine SBP to 175 mmHg compared to 150 mmHg with placebo [38].

Despite its benefits, octreotide is associated with side effects, including abdominal discomfort and gastrointestinal disturbances, which can impact patient adherence to treatment. Octreotide may be particularly beneficial for patients with refractory NOH who do not respond to other therapies. However, its requirement for parenteral administration can limit its broader use in clinical practice, as it may not be as convenient as oral medications.

The comparative summary in Table 4 describes the key characteristics of drugs used for OH, focusing on their mechanisms, highest reported improvements in SBP, symptom relief effectiveness, typical dosages, and notable side effects. Table 4 highlights these drugs’ relative strengths, making it easier to tailor treatments based on patient needs.

**Table 4:** Comparative summary of the pharmacological interventions BP: blood pressure, UTI: urinary tract infection, GI: gastrointestinal.

Drug	Mechanism	Dosage	Highest standing SBP improvement (mmHg)	Symptom relief	Notable side effects
Droxidopa	Prodrug for norepinephrine	100-600 mg, 3 times daily	+12.3	Good	Headache, dizziness, nausea
Midodrine	Alpha-1 receptor agonist	2.5-10 mg, 3 times daily	+22.4	Excellent	Pruritus, supine hypertension, urinary retention
Atomoxetine	Norepinephrine reuptake inhibitor	10-18 mg, 3 times daily	+20	Fair	Altered sensations, UTI, and upper respiratory symptoms
Pyridostigmine	Acetylcholinesterase inhibitor	30-60 mg, 3 times daily	+10	Fair	Sweating, GI disturbances, urinary urgency
Fludrocortisone	Mineralocorticoid	0.1-0.2 mg, once daily	+15	Moderate	Leg edema, headache, hypokalemia
Octreotide	Somatostatin analogue	0.5-1.0 µg/kg, subcutaneous, as needed	NA	Limited	Abdominal and GI discomfort

**Figure 3:**
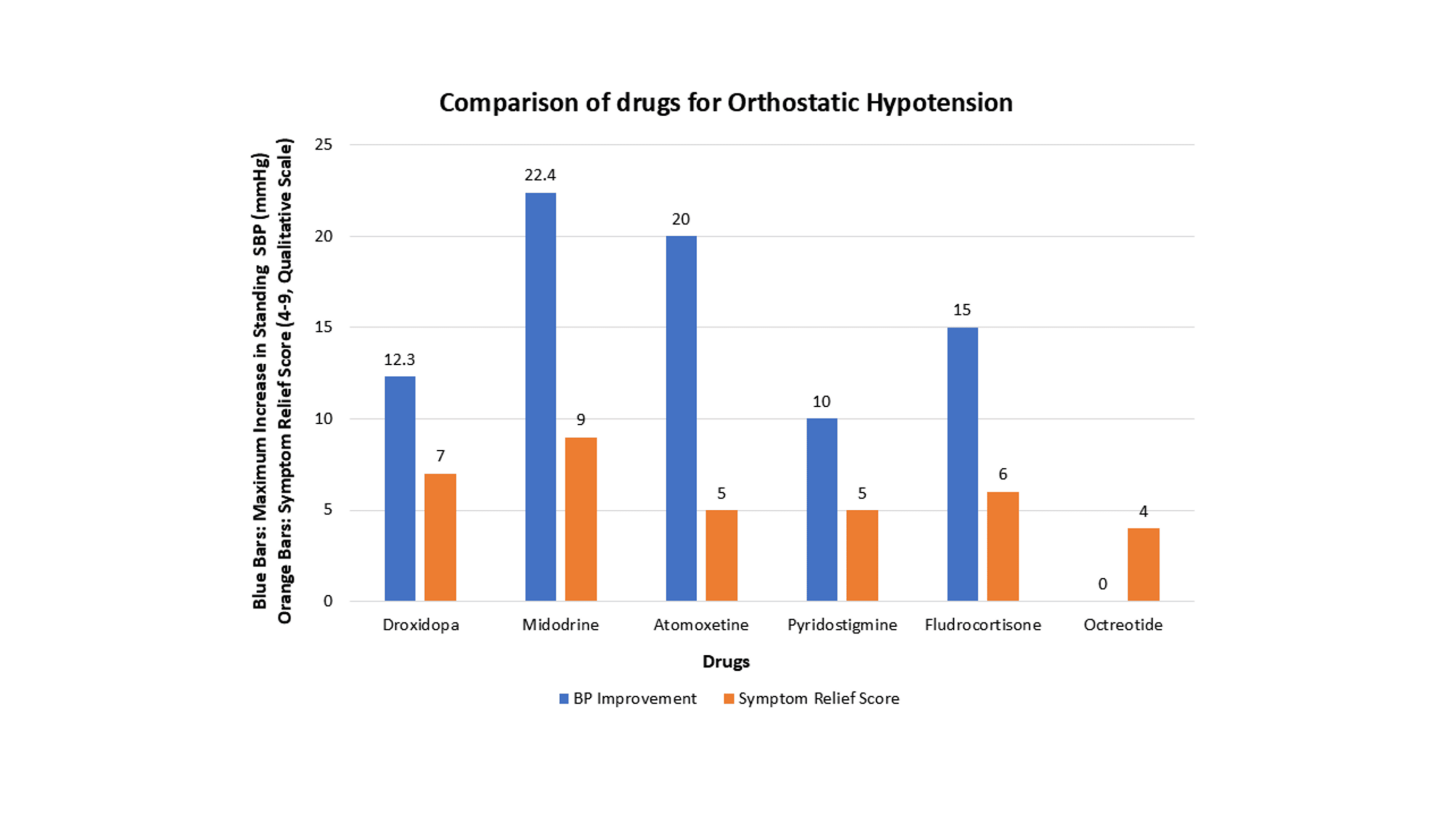
Comparison of drugs for orthostatic hypotension Blue bars represent the maximum reported improvement in standing SBP (mmHg) across studies. Data for octreotide were not available. Orange bars represent a qualitative symptom relief score (range: 4-9), derived from study-reported outcomes. Note: Data are represented as maximum values and qualitative scores, not as mean ± SD or N (%). SBP: systolic blood pressure.

Figure 3 compares the efficacy of drugs for OH based on the highest reported improvement in standing systolic blood pressure and an arbitrary symptom relief score. The blood pressure improvements were derived from the maximum values of supine or standing SBP reported in the studies within the systematic review. The arbitrary symptom relief scores were calculated qualitatively, ranging from 4 (limited relief) to 9 (excellent relief), based on data from clinical outcomes like the OHQ and patient-reported relief metrics in the study. Drugs with consistent symptom improvement across studies, such as midodrine, were assigned higher scores, while those with limited or inconsistent effects received lower scores. This approach provides a balanced visual and tabular representation of the drugs’ comparative efficacy.

The bar graph highlights that midodrine (10 mg dose) delivers a significant standing SBP improvement (+22.4 mmHg) along with excellent symptom relief, making it the most effective option overall. Atomoxetine and droxidopa offer moderate blood pressure increases (+20 mmHg and +12.3 mmHg, respectively), with droxidopa standing out for its superior symptom control. Fludrocortisone shows solid blood pressure improvement (+15 mmHg), making it ideal for chronic management through volume expansion, though its symptom relief is less pronounced. Pyridostigmine (+10 mmHg) and octreotide are less effective but can serve as valuable adjuncts or be used in specific scenarios. Overall, given the importance of symptom improvement over blood pressure control for patient benefit, midodrine, and droxidopa remain the first-line choices, while atomoxetine and fludrocortisone offer strong alternatives tailored to individual needs. However, these results may vary, as some drugs, like midodrine and droxidopa, have been studied more extensively than others.

Adverse Effects of Pharmacological Treatments in Relation to Duration of Use

The relationship between the duration of drug treatment and adverse effects for droxidopa, midodrine, atomoxetine, pyridostigmine, fludrocortisone, and octreotide indicates that adverse effects tend to increase with prolonged use. For droxidopa, short-term use (1-2 weeks) is associated with mild symptoms such as dizziness and headache, affecting approximately 18.5% of patients [15]. In contrast, long-term use (up to 12 months) significantly elevates the incidence of more serious adverse effects, including falls (20.6%) and urinary tract infections (17.6%) [17].

Midodrine exhibits a similar trend. Short-term use is typically linked to transient symptoms such as pruritus and scalp tingling [22,23]. However, extended treatment is associated with persistent side effects, including scalp pruritus (~13%), urinary urgency (~4%), and supine hypertension (~8%)[21]. For atomoxetine, a single dose generally does not result in significant side effects [29], but prolonged use can lead to increased occurrences of adverse effects, such as reflux esophagitis and upper respiratory infections, affecting up to 32.5% of users [27].

Pyridostigmine, commonly associated with gastrointestinal issues, may cause mild sweating and cramping with a single dose [32]. Over longer durations, patients may experience persistent gastrointestinal symptoms and dizziness, with an incidence of approximately 23% [33]. Fludrocortisone typically causes mild edema and headaches during treatments lasting 3-6 weeks [34]. However, chronic use is linked to a wider range of adverse effects, including hypertension, dizziness, and edema, affecting about 9% of patients [36]. Octreotide usually results in nausea and loose stools during short-term treatments [37], with gastrointestinal discomfort worsening upon repeated use.

Combination therapies, such as midodrine with pyridostigmine or octreotide, may provide symptom relief for OH but can also lead to cumulative side effects. These findings underscore the importance of careful monitoring of treatment duration to minimize adverse effects.

Certainty of Evidence

To evaluate the overall strength and reliability of the included evidence, we applied the GRADE framework [39] across five domains: risk of bias, inconsistency, indirectness, imprecision, and publication bias. Table 5 provides a structured overview of these domain-level assessments by outcome and intervention.

**Table 5:** GRADE certainty assessment by outcome and intervention PICO: In patients with orthostatic hypotension (P), do pharmacological agents (I) vs. placebo or other drugs (C) affect key outcomes (O)? OHQ: Orthostatic Hypotension Questionnaire, OHSA: Orthostatic Hypotension Symptom Assessment, RCT: randomized controlled trial, NRCT: non-randomized controlled trial, SBP: systolic blood pressure.

Outcome	Starting certainty	Domain 1: risk of bias	Domain 2: inconsistency	Domain 3: indirectness	Domain 4: imprecision	Domain 5: publication bias	Final certainty (GRADE)
Droxidopa [14-19]							
Improvement in symptoms (OHQ/OHSA)	High (RCT)	Some concerns (-1)	Serious (-1)	Not serious (0)	Not serious (0)	Not serious (0)	Low ●●○○
Standing SBP improvement	High (RCT)	Not serious (0)	Some concerns (-1)	Not serious (0)	Not serious (0)	Not serious (0)	Moderate ●●●○
Durability of response	Low (NRCT)	Serious (-1)	Not serious (0)	Not serious (0)	Not serious (0)	Not serious (0)	Very low ●○○○
Midodrine [20-25]							
Improvement in symptoms (OHQ/OHSA)	High (RCT)	Some concerns (-1)	Not serious (0)	Not serious (0)	Not serious (0)	Not serious (0)	Moderate ●●●○
Standing SBP improvement	High (RCT)	Not serious (0)	Not serious (0)	Not serious (0)	Not serious (0)	Not serious (0)	High ●●●●
Atomoxetine [26-30]							
Improvement in symptoms (OHQ/OHSA)	High (RCT)	Not serious (0)	Serious (-1)	Not serious (0)	Serious (-1)	Not serious (0)	Low ●●○○
Standing SBP improvement	High (RCT)	Not serious (0)	Not serious (0)	Not serious (0)	Some concerns (-1)	Not serious (0)	Moderate ●●●○
Pyridostigmine [25,28,31-33]							
Improvement in symptoms (OHQ/OHSA)	High (RCT)	Not serious (0)	Some concerns (-1)	Some concerns (-1)	Serious (-1)	Not serious (0)	Low ●●○○
Standing SBP improvement	High (RCT)	Not serious (0)	Some concerns (-1)	Not serious (0)	Not serious (0)	Not serious (0)	Moderate ●●●○
Fludrocortisone [34-36]							
Supine SBP improvement	Low (observational study)	Serious (-1)	Not serious (0)	Some concerns (-1)	Serious (-1)	Not serious (0)	Very low ●○○○
Durability of response	Low (Observational study)	Serious (-1)	Not serious (0)	Not serious (0)	Serious (-1)	Not serious (0)	Very low ●○○○
Octreotide [37,38]							
Standing SBP improvement	High (RCTs)	Some concerns (-1)	Serious (-1)	Not serious (0)	Serious (-1)	Not serious (0)	Very low ●○○○

Common reasons for downgrading included methodological limitations in observational studies, inconsistency in symptom-related outcomes across trials, and imprecision due to small sample sizes. Outcomes related to standing systolic blood pressure, particularly for midodrine and droxidopa demonstrated moderate to high certainty. In contrast, evidence for atomoxetine, pyridostigmine, and fludrocortisone was generally rated low or very low certainty.

Summary of Findings

The number of participants contributing to each outcome, the direction and consistency of findings, and the final GRADE certainty ratings are detailed in Table 6. This synthesis contextualizes the potential benefits of each intervention alongside the level of confidence we can place in the findings. Midodrine demonstrated high-certainty evidence for improving standing blood pressure, while droxidopa showed moderate certainty for this outcome but lower certainty for symptom improvement. Atomoxetine, fludrocortisone, and octreotide were supported by very low-certainty evidence, largely due to study design limitations and imprecision. These GRADE assessments emphasize the need for better-powered and methodologically sound trials, especially for second-line or adjunctive agents. Clinicians should interpret the reported benefits in light of the overall low-to-moderate certainty of evidence.

**Table 6:** Summary of findings (SoF) Population: adults with orthostatic hypotension. Setting: outpatient and inpatient clinical trials. Interventions: droxidopa, midodrine, atomoxetine, pyridostigmine, fludrocortisone, octreotide. Comparators: placebo or active comparator drugs. OHQ: Orthostatic Hypotension Questionnaire, OHSA: Orthostatic Hypotension Symptom Assessment, RCT: randomized controlled trial, NRCT: non-randomized controlled trial, SBP: systolic blood pressure, FD: familial dysautonomia.

Outcome	Summary	Participants (studies)	Certainty (GRADE)
Droxidopa [14-19]			
Symptom improvement (OHQ/OHSA)	Mixed results: 3 RCTs showed unclear or short-lived benefit; 1 RCT was negative; open-label study showed benefit.	707 (4 RCTs and 2 NRCTs)	Low ●●○○
Standing SBP	Transient BP increase in some RCT; effect not consistent across trials.	593 (4 RCTs and 1 NRCTs)	Moderate ●●●○
Durability of response	Open-label studies suggest sustained benefit; not verified in controlled trials.	216 (2 NRCTs)	Very low ●○○○
Midodrine [20-25]			
Symptom improvement (OHQ/OHSA)	Clear and consistent improvement in OHSA scores across RCTs.	215 (3 RCTs)	Moderate ●●●○
Standing SBP	Reliable acute pressor response noted across all trials.	510 (6 RCTs)	High ●●●●
Atomoxetine [26-30]			
Symptom improvement (OHQ/OHSA)	Conflicting findings; small samples and short durations limit certainty.	211 (5 RCTs)	Very low ●○○○
Standing SBP	Pressor response reproducible in most studies.	161 (4 RCTs)	Moderate ●●●○
Pyridostigmine [25,28,31-33]			
Symptom improvement (OHQ/OHSA)	Modest benefit seen; studies are heterogeneous and underpowered.	112 (3 RCTs)	Low ●●○○
Standing SBP	Modest but consistent increase in SBP observed.	185 (4 RCTs and 1 NRCT)	Moderate ●●●○
Fludrocortisone [34-36]			
Supine SBP	Inconsistent effects across small, uncontrolled studies.	19 (2 RCTs)	Very low ●○○○
Durability	Some long-term benefit in FD reported; limited generalizability.	341 (1 observational study)	Very low ●○○○
Octreotide [37,38]			
Standing SBP	Acute SBP improvement in small crossover studies; no long-term data or symptom scores reported.	25 (2 RCTs)	Very low ●○○○

Discussion

This systematic review synthesized evidence from 25 studies investigating the pharmacological management of OH caused by neurogenic conditions. Our findings confirm that several agents are effective in improving both hemodynamic measures and clinical symptoms. Although the quality and consistency of evidence vary, the GRADE approach revealed that some findings, such as midodrine’s effect on SBP, are supported by high or moderate certainty. In contrast, other outcomes, particularly those from small trials or observational studies, were rated as low or very low certainty. The alpha-1 adrenergic agonist midodrine and the norepinephrine prodrug droxidopa emerged as the most robustly supported first-line therapies. Other medications, including atomoxetine and fludrocortisone, demonstrated moderate efficacy, while agents like pyridostigmine and octreotide may serve as valuable adjunctive therapies. This discussion will interpret these principal findings, compare and contrast the evidence within our included studies, place them in the context of existing literature, address the clinical implications and limitations of this review, and propose directions for future research.

A deeper analysis of our included studies reveals important nuances in the evidence for the leading agents, droxidopa and midodrine. While both drugs demonstrated efficacy, the nature of their supporting evidence differs. The trials on midodrine, such as those by Low et al. [20] and Jankovic et al. [21], reported the most dramatic improvements in standing SBP (up to 22 mmHg), establishing its potent pressor effect. However, these benefits were often accompanied by a significant risk of supine hypertension and bothersome side effects like pruritus. In contrast, the evidence for droxidopa, particularly from large-scale studies like Kaufmann et al. [15] and Hauser et al. [14], emphasized a more modest but clinically meaningful improvement in SBP (7-12 mmHg) coupled with significant gains in patient-reported symptoms via the OHQ. This suggests a potential dissociation between the magnitude of blood pressure change and symptomatic relief, where droxidopa’s primary value may lie in improving daily function with a lower risk of iatrogenic supine hypertension. This trade-off between potent hemodynamic effect and symptomatic benefit with better tolerability is a central theme emerging from our synthesis.

Furthermore, the evidence for second-line and adjunctive therapies highlights the importance of mechanism-based treatment selection. The studies on atomoxetine, a norepinephrine reuptake inhibitor, produced conflicting results. While Ramirez et al. [29] found its pressor effect superior to midodrine, two other RCTs [26,27] failed to show a significant benefit over placebo. This heterogeneity may reflect differences in patient populations; atomoxetine is theorized to be more effective in patients with preserved sympathetic neuronal function. Similarly, the utility of pyridostigmine was most apparent when used as an adjunct. The study by Singer et al. [31] showed that while it offered minimal SBP benefit alone, it significantly augmented the pressor response to midodrine, likely by enhancing ganglionic cholinergic neurotransmission. This contrasts with the volume-expanding mechanism of fludrocortisone, which demonstrated consistent, moderate efficacy in chronic management but carries a distinct side effect profile of edema and hypokalemia [34-36]. Together, these findings illustrate that beyond a simple hierarchy of efficacy, the optimal treatment strategy likely involves selecting agents whose mechanisms target the patient's specific underlying pathophysiology.

The application of the GRADE framework adds a valuable layer to our interpretation of findings. While midodrine and droxidopa are supported by moderate-to-high certainty for blood pressure outcomes, other agents such as atomoxetine, pyridostigmine, and fludrocortisone received low to very low ratings due to imprecision, risk of bias, and inconsistent effects. These assessments highlight the need for stronger evidence before broader clinical endorsement of these adjunctive treatments. Moreover, the observed downgrades reinforce the importance of cautious interpretation of symptomatic outcomes, which often showed greater heterogeneity and subjectivity.

Comparative Analysis With Existing Literature

Our study builds upon the findings of previous systematic reviews, specifically those by Ong et al. [48] and Kulkarni et al. [49], both of whom explored pharmacological treatments for OH. While Ong et al. [48] included 13 RCTs focusing primarily on midodrine and fludrocortisone, Kulkarni et al. [49] expanded their review to 19 RCTs, examining a broader array of medications, including midodrine, atomoxetine, and pyridostigmine, and conducted a meta-analysis on six studies.

A key distinction between these studies and ours lies in the methodological approach. Ong et al. [48] relied on descriptive summaries without performing a meta-analysis, resulting in a lack of data uniformity. In contrast, Kulkarni et al. (2022) [49] quantified changes in systolic blood pressure (SBP) but focused on a narrower set of outcomes. Our study, however, examines both short- and long-term outcomes (up to 12 months) of SBP, along with associated symptoms, while also evaluating a broader range of pharmacological interventions, thus providing a more comprehensive perspective.

Furthermore, while Ong et al. [48] concentrated on earlier studies with limited outcome diversity, concluding that midodrine and fludrocortisone effectively increase SBP in specific patient populations, their findings were constrained by inconsistent data and suboptimal clinical trial designs [48]. In contrast, our research emphasizes standardized measures, such as SBP and the OHQ, reflecting a contemporary approach to assessing treatment efficacy. We address the limitations identified in the work of Ong et al. [48] by including only high-quality studies and employing robust quality appraisal tools.

Kulkarni et al. [49] identified midodrine as the most impactful treatment among the evaluated drugs, a finding consistent with our results, which also affirm midodrine's reliability in significantly improving SBP and alleviating orthostatic symptoms. Additionally, our study highlights the benefits of droxidopa in managing OH symptoms and thoroughly evaluates the adverse effects of all interventions, incorporating both short- and long-term outcomes.

Finally, we underscore the variability in individual responses to treatment and the critical need for standardized outcome measures. This aligns with Kulkarni et al. [49], who reported significant heterogeneity in trial designs and emphasized the importance of long-term studies to enhance clinical applicability. Overall, our findings contribute to a more nuanced understanding of pharmacological interventions for OH, addressing previous gaps in the literature.

Clinical Implication

The findings from this systematic review provide healthcare professionals with a comprehensive overview of the efficacy and safety of various pharmacological interventions for managing OH. These clinical implications must be considered alongside the certainty of the underlying evidence, as determined by our GRADE assessment. By synthesizing evidence from multiple studies, clinicians can make informed decisions about which medications, such as droxidopa, midodrine, fludrocortisone, atomoxetine, and pyridostigmine, are most effective for their patients. This knowledge is particularly crucial for tailoring treatment plans to individual patients based on the underlying cause of OH, comorbidities, and concurrent medications.

In terms of blood pressure improvement, midodrine demonstrated the most consistent and significant effects, making it the superior choice for acute management. For symptom control, both midodrine and droxidopa were effective, though midodrine held a slight advantage based on patient-reported relief. Fludrocortisone was most effective for volume expansion but requires careful monitoring due to the risk of hypokalemia. While atomoxetine showed strong blood pressure improvement, its results lacked consistent statistical significance across studies. Although pyridostigmine and octreotide demonstrated lower overall effectiveness, their niche benefits make them valuable as adjuncts or secondary treatments in specific scenarios.

Effective management of OH is essential to reduce debilitating symptoms such as dizziness, syncope, and falls, which can lead to significant morbidity, including fractures and hospitalizations. The review highlights that pharmacological treatments can significantly improve blood pressure stability and alleviate symptoms, thereby enhancing patients' quality of life. Clinicians should prioritize early identification and treatment of OH, particularly in vulnerable populations such as the elderly and those with neurodegenerative diseases, to mitigate the associated risks of functional impairment and increased mortality.

The variability in treatment responses observed across studies underscores the importance of personalized medicine in managing OH. Clinicians should consider factors such as age, underlying health conditions, and specific symptoms when selecting pharmacological treatments. The review suggests that combination therapies may also be beneficial for some patients, indicating a need for further research into optimal treatment regimens tailored to individual patient profiles.

The effectiveness of different treatments for OH can vary significantly due to the diverse underlying causes of the condition, as well as the distinct mechanisms through which medications operate. Therefore, treatment selection should take into account the patient’s underlying pathophysiology. For instance, patients with degeneration of peripheral noradrenergic neurons and low plasma norepinephrine levels, such as those with pure autonomic failure or PD, generally respond better to drugs like midodrine or droxidopa, which directly or indirectly restore norepinephrine levels [50]. Conversely, individuals with relatively preserved peripheral sympathetic function and normal or slightly reduced plasma norepinephrine levels, as observed in multiple system atrophy, are more likely to benefit from medications like pyridostigmine or atomoxetine, which enhance the effects of norepinephrine [50].

Given the complexity of OH and its underlying causes, a multidisciplinary approach to management is essential. Collaboration among primary care physicians, neurologists, geriatricians, and pharmacists can ensure comprehensive care that addresses the multifaceted nature of the condition. Education and training for healthcare providers about the latest evidence-based pharmacological interventions can enhance clinical practice and improve patient outcomes.

Strengths and Limitations of the Included Studies

The studies included in this systematic review exhibit several strengths that enhance the credibility of the findings. Notably, many of these studies feature adequate sample sizes, which bolster the statistical power of the results. Larger sample sizes facilitate more reliable estimates of treatment effects, thereby minimizing the risk of random error. Additionally, the diversity of populations represented in the studies, including variations in age and comorbidities, supports the generalizability of the findings to a broader demographic of patients with OH. Furthermore, several studies directly compare multiple pharmacological interventions, providing valuable insights into the relative effectiveness of treatments across different sample sizes.

Despite these strengths, the included studies also present notable limitations. Some studies have small sample sizes, which can limit the reliability of their findings and increase the potential for type I and type II errors. Moreover, the variability in sample characteristics across studies may introduce confounding factors that complicate the interpretation of results. Furthermore, in certain studies, participants were found to be utilizing non-pharmacological management strategies alongside the intended pharmacological treatments. This concurrent use of interventions may influence the outcomes, making it challenging to ascertain the true efficacy of the primary treatment being evaluated. These limitations highlight the need for caution in generalizing the findings and underscore the importance of further research with larger, more homogeneous samples to enhance the robustness of conclusions drawn in this field.

Strengths and Limitations of the Review Process

The systematic review process demonstrates several strengths that contribute to its overall rigor. A major strength is the inclusion of 25 studies, with 20 of these being RCTs, which is considered very robust. This substantial representation of RCTs enhances the statistical power of our results, providing strong evidence for the efficacy of pharmacological interventions in managing OH. Additionally, the inclusion of diverse studies with sample sizes varying from 6 to 341 allows for a comprehensive understanding of pharmacological interventions in managing OH. The rigorous selection criteria employed during the review ensured that only high-quality studies were included, which enhances the credibility of the findings. Moreover, adherence to PRISMA 2020 guidelines provided a structured and transparent approach to the review process, further reinforcing the validity of the conclusions drawn.

However, the review process is not without its limitations. One significant constraint is the exclusion of non-English studies, which may have resulted in the omission of relevant research published in other languages. This limitation potentially narrows the evidence base and impacts the comprehensiveness of the review. Additionally, access limitations to certain studies due to paywalls or publication restrictions may have prevented their inclusion, introducing selection bias into the review. Furthermore, while many studies had adequate sample sizes, the presence of smaller studies could affect the overall strength of the conclusions drawn from the review.

Future Research

The findings from this systematic review highlight several critical gaps in the current understanding of pharmacological interventions for OH. Future research should prioritize large-scale, multi-center randomized controlled trials to evaluate the long-term efficacy and safety of existing treatments, particularly focusing on patient-reported outcomes and quality of life measures. While treatments such as midodrine and droxidopa demonstrate clear benefits in increasing standing systolic blood pressure and alleviating symptoms, the effectiveness of newer medications like atomoxetine requires further investigation to fully understand their role in managing OH. Future studies should also adhere to GRADE principles by clearly reporting predefined outcomes, minimizing bias, and including sufficient power to reduce imprecision in effect estimates.

Additionally, there is a pressing need for studies that explore the effects of combination therapies tailored to specific patient profiles, as this could enhance treatment efficacy and minimize adverse effects. Investigating the pharmacogenomics of OH treatments may also provide insights into individualized therapy, allowing clinicians to optimize drug selection based on genetic predispositions. Moreover, exploring non-pharmacological interventions in conjunction with pharmacotherapy could yield comprehensive management strategies that address the multifactorial nature of OH.

## Conclusions

This systematic review highlights the critical role of pharmacological interventions in the management of OH, particularly among vulnerable populations such as the elderly and those with neurodegenerative disorders. The evidence demonstrates that medications like droxidopa and midodrine are effective first-line treatments, prioritizing symptom alleviation, which significantly enhances patient quality of life, alongside improving standing blood pressure. However, the certainty of evidence across interventions varied, as evaluated using the GRADE framework. While findings for midodrine and droxidopa were supported by moderate to high certainty for hemodynamic outcomes, the evidence for other agents was frequently limited by risk of bias, small sample sizes, and inconsistent effects.

Moreover, the review identifies important gaps in the current literature, particularly regarding the long-term efficacy and safety of newer agents like atomoxetine, as well as the potential benefits of combination therapies. Future research should prioritize large-scale, multicenter RCTs to address these gaps and explore the pharmacogenomics of treatment options. By enhancing our understanding of individualized medicine and integrating non-pharmacological strategies, we can optimize therapeutic outcomes and reduce symptom burden in patients suffering from this debilitating condition.

## Appendices

**Table 7:** Search strategy EBSCO: Elton B. Stephens Company.

Search strategy	Database/register searched	No. of papers before I/E criteria application	Filters used	No. of papers after filter application	Date searched
("hypotension, orthostatic"[MeSH Terms] OR ("hypotension"[All Fields] AND "orthostatic"[All Fields]) OR "orthostatic hypotension"[All Fields] OR ("orthostatic"[All Fields] AND "hypotension"[All Fields]) OR ("hypotension, orthostatic"[MeSH Terms] OR ("hypotension""[All Fields] AND "orthostatic"[All Fields]) OR "orthostatic hypotension"[All Fields] OR ("postural"[All Fields] AND "hypotension"[All Fields]) OR "postural hypotension"[All Fields]) OR "hypotension, orthostatic"[MeSH Terms] OR ("hypotension, orthostatic/drug therapy"[MeSH Terms] OR "hypotension, orthostatic/therapy"[MeSH Terms])) AND ("midodrine"[MeSH Terms] OR "midodrine"[All Fields] OR "midodrin"[All Fields] OR ("midodrine"[MeSH Terms] OR "midodrine"[All Fields] OR "midodrin"[All Fields]) OR ("midodrine"[MeSH Terms] OR "midodrine"[All Fields] OR ("midodrine"[All Fields] AND "hydrochloride"[All Fields]) OR "midodrine hydrochloride"[All Fields]) OR ("midodrine"[MeSH Terms] OR "midodrine"[All Fields] OR ("midodrine"[All Fields] AND "monohydrochloride"[All Fields])) OR ("adrenergic alpha 1 receptor agonists"[Pharmacological Action] OR "adrenergic alpha 1 receptor agonists"[MeSH Terms] OR ("adrenergic"[All Fields] AND "alpha 1"[All Fields] AND "receptor"[All Fields] AND "agonists"[All Fields]) OR "adrenergic alpha 1 receptor agonists"[All Fields] OR "adrenergic alpha 1 receptor agonists"[All Fields]) OR ("vasoconstrictor agents"[Pharmacological Action] OR "vasoconstrictor agents"[MeSH Terms] OR ("vasoconstrictor"[All Fields] AND "agents"[All Fields]) OR "vasoconstrictor agents"[All Fields]) OR "midodrine/adverse effects"[MeSH Terms] OR "midodrine/therapeutic use"[MeSH Terms] OR ("adrenergic alpha 1 receptor agonists/adverse effects"[MeSH Terms] OR "adrenergic alpha 1 receptor agonists/therapeutic use"[MeSH Terms]) OR ("vasoconstrictor agents/adverse effects"[MeSH Terms] OR "vasoconstrictor agents/therapeutic use"[MeSH Terms]) OR ("droxidopa"[MeSH Terms] OR "droxidopa"[All Fields] OR ("antiparkinson agents"[Pharmacological Action] OR "antiparkinson agents"[MeSH Terms] OR ("antiparkinson"[All Fields] AND "agents"[All Fields]) OR "antiparkinson agents"[All Fields] OR ("antiparkinson"[All Fields] AND "drugs"[All Fields]) OR "antiparkinson drugs"[All Fields]) OR ("antiparkinson agents"[Pharmacological Action] OR "antiparkinson agents"[MeSH Terms] OR ("antiparkinson"[All Fields] AND "agents"[All Fields]) OR "antiparkinson agents"[All Fields] OR ("antiparkinsonian"[All Fields] AND "agents"[All Fields]) OR "antiparkinsonian agents"[All Fields]) OR ("antiparkinson agents"[Pharmacological Action] OR "antiparkinson agents"[MeSH Terms] OR ("antiparkinson"[All Fields] AND "agents"[All Fields]) OR "antiparkinson agents"[All Fields] OR "antiparkinsonian"[All Fields] OR "antiparkinsonians"[All Fields]) OR ("droxidopa/adverse effects"[MeSH Terms] OR "droxidopa/therapeutic use"[MeSH Terms]) OR ("antiparkinson agents/adverse effects"[MeSH Terms] OR "antiparkinson agents/therapeutic use"[MeSH Terms])) OR ("fludrocortisone"[MeSH Terms] OR "fludrocortisone"[All Fields] OR "fludrocortisones"[All Fields] OR ("fludrocortisone/adverse effects"[MeSH Terms] OR "fludrocortisone/therapeutic use"[MeSH Terms])) OR ("octreotid"[All Fields] OR "octreotide"[MeSH Terms] OR "octreotide"[All Fields] OR "octreotides"[All Fields] OR ("octreotide/adverse effects"[MeSH Terms] OR "octreotide/therapeutic use"[MeSH Terms])) OR ("atomoxetine hydrochloride"[MeSH Terms] OR ("atomoxetine"[All Fields] AND "hydrochloride"[All Fields]) OR "atomoxetine hydrochloride"[All Fields] OR "atomoxetine"[All Fields] OR "atomoxetine s"[All Fields] OR ("atomoxetine hydrochloride"[MeSH Terms] OR ("atomoxetine"[All Fields] AND "hydrochloride"[All Fields]) OR "atomoxetine hydrochloride"[All Fields] OR ("atomoxetine"[All Fields] AND "hcl"[All Fields]) OR "atomoxetine hcl"[All Fields]) OR ("atomoxetine hydrochloride"[MeSH Terms] OR ("atomoxetine"[All Fields] AND "hydrochloride"[All Fields]) OR "atomoxetine hydrochloride"[All Fields] OR "tomoxetine"[All Fields]) OR ("adrenergic uptake inhibitors"[Pharmacological Action] OR "adrenergic uptake inhibitors"[MeSH Terms] OR ("adrenergic"[All Fields] AND "uptake"[All Fields] AND "inhibitors""[All Fields]) OR "adrenergic uptake inhibitors"[All Fields] OR ("adrenergic"[All Fields] AND "reuptake"[All Fields] AND "inhibitors"[All Fields]) OR "adrenergic reuptake inhibitors"[All Fields]) OR ("atomoxetine hydrochloride/adverse effects"[MeSH Terms] OR "atomoxetine hydrochloride/therapeutic use"[MeSH Terms]) OR ("adrenergic uptake inhibitors/adverse effects"[MeSH Terms] OR "adrenergic uptake inhibitors/therapeutic use"[MeSH Terms])) OR ("pyridostigmin"[All Fields] OR "pyridostigmine bromide"[MeSH Terms] OR ("pyridostigmine"[All Fields] AND "bromide"[All Fields]) OR "pyridostigmine bromide"[All Fields] OR "pyridostigmine"[All Fields] OR ("anticholinesterasic"[All Fields] OR "anticholinesterasics"[All Fields] OR "cholinesterase inhibitors"[Pharmacological Action] OR "cholinesterase inhibitors"[MeSH Terms] OR ("cholinesterase"[All Fields] AND "inhibitors"[All Fields]) OR "cholinesterase inhibitors"[All Fields] OR "anticholinesterase"[All Fields] OR "anticholinesterases"[All Fields]) OR ("cholinesterase inhibitors"[Pharmacological Action] OR "cholinesterase inhibitors"[MeSH Terms] OR ("cholinesterase"[All Fields] AND "inhibitors"[All Fields]) OR "cholinesterase inhibitors"[All Fields] OR ("acetylcholinesterase"[All Fields] AND "inhibitors"[All Fields]) OR "acetylcholinesterase inhibitors"[All Fields]) OR ("cholinesterase inhibitors"[Pharmacological Action] OR "cholinesterase inhibitors"[MeSH Terms] OR ("cholinesterase"[All Fields] AND "inhibitors"[All Fields]) OR "cholinesterase inhibitors"[All Fields]) OR ("pyridostigmine bromide/adverse effects"[MeSH Terms] OR "pyridostigmine bromide/therapeutic use"[MeSH Terms]) OR ("cholinesterase inhibitors/adverse effects"[MeSH Terms] OR "cholinesterase inhibitors/therapeutic use"[MeSH Terms])) OR ("Drug Therapy"[MeSH Terms] OR ("drug"[All Fields] AND "therapy"[All Fields]) OR "Drug Therapy"[All Fields] OR "pharmacotherapies"[All Fields] OR "Drug Therapy"[MeSH Subheading] OR "pharmacotherapy"[All Fields] OR ("Drug Therapy"[MeSH Terms] OR ("drug"[All Fields] AND "therapy"[All Fields]) OR "Drug Therapy"[All Fields] OR "pharmacotherapies"[All Fields] OR "Drug Therapy"[MeSH Subheading] OR "pharmacotherapy"[All Fields]) OR ("Drug Therapy"[MeSH Terms] OR ("drug"[All Fields] AND "therapy"[All Fields]) OR "Drug Therapy"[All Fields] OR ("drug"[All Fields] AND "therapies"[All Fields]) OR "drug therapies"[All Fields]) OR ("Drug Therapy"[MeSH Subheading] OR ("drug"[All Fields] AND "therapy"[All Fields]) OR "Drug Therapy"[All Fields] OR "Drug Therapy"[MeSH Terms]) OR ("cardiovascular agents"[Pharmacological Action] OR "cardiovascular agents"[MeSH Terms] OR ("cardiovascular"[All Fields] AND "agents"[All Fields]) OR "cardiovascular agents"[All Fields] OR ("cardiovascular"[All Fields] AND "drug""[All Fields]) OR "cardiovascular drug"[All Fields]) OR ("cardiovascular agents"[Pharmacological Action] OR "cardiovascular agents"[MeSH Terms] OR ("cardiovascular"[All Fields] AND "agents"[All Fields]) OR "cardiovascular agents"[All Fields] OR ("cardiovascular"[All Fields] AND "drugs""[All Fields]) OR "cardiovascular drugs"[All Fields]) OR ("cardiovascular agents"[Pharmacological Action] OR "cardiovascular agents"[MeSH Terms] OR ("cardiovascular"[All Fields] AND "agents"[All Fields]) OR "cardiovascular agents"[All Fields] OR ("cardiovascular"[All Fields] AND "agent"[All Fields]) OR "cardiovascular agent"[All Fields]) OR ("cardiovascular agents"[Pharmacological Action] OR "cardiovascular agents"[MeSH Terms] OR ("cardiovascular"[All Fields] AND "agents"[All Fields]) OR "cardiovascular agents"[All Fields]) OR ("cardiovascular agents"[Pharmacological Action] OR "cardiovascular agents"[MeSH Terms] OR ("cardiovascular"[All Fields] AND "agents"[All Fields]) OR "cardiovascular agents"[All Fields] OR ("cardioactive"[All Fields] AND ""agent"[All Fields]) OR "cardioactive agent"[All Fields]) OR ("cardiovascular agents"[Pharmacological Action] OR "cardiovascular agents"[MeSH Terms] OR ("cardiovascular"[All Fields] AND "agents"[All Fields]) OR "cardiovascular agents"[All Fields] OR ("cardioactive"[All Fields] AND "drug"[All Fields]) OR "cardioactive drug"[All Fields]) OR ("cardiovascular agents"[Pharmacological Action] OR "cardiovascular agents"[MeSH Terms] OR ("cardiovascular"[All Fields] AND "agents"[All Fields]) OR "cardiovascular agents"[All Fields] OR ("cardioactive"[All Fields] AND "agents"[All Fields]) OR "cardioactive agents"[All Fields]) OR ("cardiovascular agents"[Pharmacological Action] OR "cardiovascular agents"[MeSH Terms] OR ("cardiovascular"[All Fields] AND "agents"[All Fields]) OR "cardiovascular agents"[All Fields] OR ("cardioactive"[All Fields] AND "drugs"[All Fields]) OR "cardioactive drugs"[All Fields]) OR "Drug Therapy"[MeSH Terms] OR ("cardiovascular agents/adverse effects"[MeSH Terms] OR "cardiovascular agents/therapeutic use"[MeSH Terms])))"	PubMed/Medline	4944	Free full text, full text, clinical study, clinical trial, randomized controlled trial, observational studies in English, humans	176	11/16/2024
#1 ‘’Orthostatic hypotension’’ OR ‘’Postural Hypotension’’ 2565 #2 MeSH descriptor: [Hypotension, Orthostatic] explode all trees 543 #3 #1 OR #2 2565 #4 Midodrine OR Midodrin OR ‘’Midodrine Hydrochloride’’ OR ‘’Midodrine Monohydrochloride’’ OR ‘’Adrenergic alpha-1 Receptor Agonists’’ OR ‘’Vasoconstrictor Agents’’ 4447 #5 MeSH descriptor: [Midodrine] explode all trees 177 #6 MeSH descriptor: [Adrenergic alpha-1 Receptor Agonists] explode all trees 55 #7 MeSH descriptor: [Vasoconstrictor Agents] explode all trees 2304 #8 Droxidopa OR ‘’Antiparkinson Drugs’’ OR ‘’Antiparkinsonian Agents’’ OR Antiparkinsonians 647 #9 MeSH descriptor: [Droxidopa] explode all trees 61 #10 MeSH descriptor: [Antiparkinson Agents] explode all trees 1321 #11 Fludrocortisone 320 #12 MeSH descriptor: [Fludrocortisone] explode all trees 151 #13 Octreotide 1782 #14 MeSH descriptor: [Octreotide] explode all trees 888 #15 Atomoxetine OR ‘’Atomoxetine HCl’’ OR Tomoxetine OR ‘’Adrenergic Reuptake Inhibitors’’ 1301 #16 MeSH descriptor: [Atomoxetine Hydrochloride] explode all trees 464 #17 MeSH descriptor: [Adrenergic Uptake Inhibitors] explode all trees 595 #18 Pyridostigmine OR ‘’Anticholinesterases’’ OR ''Acetylcholinesterase Inhibitors'' OR ''Cholinesterase Inhibitors'' 2166 #19 MeSH descriptor: [Pyridostigmine Bromide] explode all trees 217 #20 MeSH descriptor: [Cholinesterase Inhibitors] explode all trees 1235 #21 Pharmacotherapy OR Pharmacotherapies OR ''Drug Therapies'' OR ''Drug Therapy'' OR ''Cardiovascular Drug'' OR ''Cardiovascular Drugs'' OR ''Cardiovascular Agent'' OR ''Cardiovascular Agents'' OR ''Cardioactive Agent'' OR ''Cardioactive Drug'' OR ''Cardioactive Agents'' OR ''Cardioactive Drugs'' 595163 #22 MeSH descriptor: [Drug Therapy] explode all trees 186928 #23 MeSH descriptor: [Cardiovascular Agents] explode all trees 29560 #24 #4 OR #5 OR #6 OR #7 OR #8 OR #9 OR #10 OR #11 OR #12 OR #13 OR #14 OR #15 OR #16 OR #17 OR #18 OR #19 OR #20 OR #21 OR #22 OR #23 645897 #25 #3 AND #24 1888	Cochrane Library	1888	Article type: trials. Languages: English	1566	11/15/2024
Search 1: Title, abstract or author-specified keywords (‘’Orthostatic hypotension’’ OR ‘’Postural Hypotension’’) AND (Midodrine OR ‘’Adrenergic alpha-1 Receptor Agonists’’ OR ‘’Vasoconstrictor Agents’’ OR Droxidopa OR ‘’Antiparkinson Drugs’’ OR Fludrocortisone OR Octreotide) Search 2: Title, abstract or author-specified keywords (‘’Orthostatic hypotension’’ OR ‘’Postural Hypotension’’) AND (Atomoxetine OR ‘’Adrenergic Reuptake Inhibitors’’ OR Pyridostigmine OR ''Acetylcholinesterase Inhibitors'' OR Pharmacotherapy OR ''Drug Therapy'' OR ''Cardiovascular Agent'')	ScienceDirect	22	Article type: research articles. Languages: English. Access type: open access & open archive	9	11/16/2024
(‘’Orthostatic hypotension’’ OR ‘’Postural Hypotension’’) AND (Midodrine OR Midodrin OR ‘’Midodrine Hydrochloride’’ OR ‘’Midodrine Monohydrochloride’’ OR ‘’Adrenergic alpha-1 Receptor Agonists’’ OR ‘’Vasoconstrictor Agents’’ OR Droxidopa OR ‘’Antiparkinson Drugs’’ OR ‘’Antiparkinsonian Agents’’ OR Antiparkinsonians OR Fludrocortisone OR Octreotide OR Atomoxetine OR ‘’Atomoxetine HCl’’ OR Tomoxetine OR ‘’Adrenergic Reuptake Inhibitors’’ OR Pyridostigmine OR ‘’Anticholinesterases’’ OR ''Acetylcholinesterase Inhibitors'' OR ''Cholinesterase Inhibitors'' OR Pharmacotherapy OR Pharmacotherapies OR ''Drug Therapies'' OR ''Drug Therapy'' OR ''Cardiovascular Drug'' OR ''Cardiovascular Drugs'' OR ''Cardiovascular Agent'' OR ''Cardiovascular Agents'' OR ''Cardioactive Agent'' OR ''Cardioactive Drug'' OR ''Cardioactive Agents'' OR ''Cardioactive Drugs'') AND (((SRC:MED OR SRC:PMC OR SRC:AGR OR SRC:CBA) NOT (PUB_TYPE:"Review"))) AND (HAS_FT:Y) AND (((SRC:MED OR SRC:PMC OR SRC:AGR OR SRC:CBA) NOT (PUB_TYPE:"Review")))	Europe PMC	296	Article type: research articles. Free full-text access: full text in Europe PMC	295	11/15/2024
‘’Orthostatic hypotension’’ OR ‘’Postural Hypotension’’ | Midodrine OR Midodrin OR ‘’Midodrine Hydrochloride’’ OR ‘’Midodrine Monohydrochloride’’ OR ‘’Adrenergic alpha-1 Receptor Agonists’’ OR ‘’Vasoconstrictor Agents’’ OR Droxidopa OR ‘’Antiparkinson Drugs’’ OR ‘’Antiparkinsonian Agents’’ OR Antiparkinsonians OR Fludrocortisone OR Octreotide OR Atomoxetine OR ‘’Atomoxetine HCl’’ OR Tomoxetine OR ‘’Adrenergic Reuptake Inhibitors’’ OR Pyridostigmine OR ‘’Anticholinesterases’’ OR ''Acetylcholinesterase Inhibitors'' OR ''Cholinesterase Inhibitors'' OR Pharmacotherapy OR Pharmacotherapies OR ''Drug Therapies'' OR ''Drug Therapy'' OR ''Cardiovascular Drug'' OR ''Cardiovascular Drugs'' OR ''Cardiovascular Agent'' OR ''Cardiovascular Agents'' OR ''Cardioactive Agent'' OR ''Cardioactive Drug'' OR ''Cardioactive Agents'' OR ''Cardioactive Drugs'' | Completed studies | Interventional, Observational studies | Studies with results	ClinicalTrials.gov	66	Completed studies, interventional, observational studies, studies with results	19	11/16/2024
(‘’Orthostatic hypotension’’ OR ‘’Postural Hypotension’’) AND (Midodrine OR Midodrin OR ‘’Midodrine Hydrochloride’’ OR ‘’Midodrine Monohydrochloride’’ OR ‘’Adrenergic alpha-1 Receptor Agonists’’ OR ‘’Vasoconstrictor Agents’’ OR Droxidopa OR ‘’Antiparkinson Drugs’’ OR ‘’Antiparkinsonian Agents’’ OR Antiparkinsonians OR Fludrocortisone OR Octreotide OR Atomoxetine OR ‘’Atomoxetine HCl’’ OR Tomoxetine OR ‘’Adrenergic Reuptake Inhibitors’’ OR Pyridostigmine OR ‘’Anticholinesterases’’ OR ''Acetylcholinesterase Inhibitors'' OR ''Cholinesterase Inhibitors'' OR Pharmacotherapy OR Pharmacotherapies OR ''Drug Therapies'' OR ''Drug Therapy'' OR ''Cardiovascular Drug'' OR ''Cardiovascular Drugs'' OR ''Cardiovascular Agent'' OR ''Cardiovascular Agents'' OR ''Cardioactive Agent'' OR ''Cardioactive Drug'' OR ''Cardioactive Agents'' OR ''Cardioactive Drugs'')	International Clinical Trials Registry Platform (ICTRP)	57	With results only	25	11/16/2024
(‘’Orthostatic hypotension’’ OR ‘’Postural Hypotension’’) AND (Midodrine OR Midodrin OR ‘’Midodrine Hydrochloride’’ OR ‘’Midodrine Monohydrochloride’’ OR ‘’Adrenergic alpha-1 Receptor Agonists’’ OR ‘’Vasoconstrictor Agents’’ OR Droxidopa OR ‘’Antiparkinson Drugs’’ OR ‘’Antiparkinsonian Agents’’ OR Antiparkinsonians OR Fludrocortisone OR Octreotide OR Atomoxetine OR ‘’Atomoxetine HCl’’ OR Tomoxetine OR ‘’Adrenergic Reuptake Inhibitors’’ OR Pyridostigmine OR ‘’Anticholinesterases’’ OR ''Acetylcholinesterase Inhibitors'' OR ''Cholinesterase Inhibitors'' OR Pharmacotherapy OR Pharmacotherapies OR ''Drug Therapies'' OR ''Drug Therapy'' OR ''Cardiovascular Drug'' OR ''Cardiovascular Drugs'' OR ''Cardiovascular Agent'' OR ''Cardiovascular Agents'' OR ''Cardioactive Agent'' OR ''Cardioactive Drug'' OR ''Cardioactive Agents'' OR ''Cardioactive Drugs'')	EBSCO open dissertations	4	No filters	4	11/16/2024
